# A regression discontinuity analysis of the social distancing recommendations for older adults in Sweden during COVID-19

**DOI:** 10.1093/eurpub/ckac101

**Published:** 2022-08-13

**Authors:** Carl Bonander, Debora Stranges, Johanna Gustavsson, Matilda Almgren, Malin Inghammar, Mahnaz Moghaddassi, Anton Nilsson, Joan Capdevila Pujol, Claire Steves, Paul W Franks, Maria F Gomez, Tove Fall, Jonas Björk, COVID Symptom Study Sweden

**Affiliations:** Health Economics & Policy, School of Public Health & Community Medicine, Sahlgrenska Academy, University of Gothenburg, Sweden; Division of Occupational and Environmental Medicine, Lund University, Lund, Sweden; Centre for Societal Risk Research, Karlstad University, Sweden; Clinical Studies Sweden, Forum South, Skåne University Hospital, Lund, Sweden; Department of Clinical Sciences Lund, Section for Infection Medicine, Skåne University Hospital, Lund University, Lund, Sweden; Social Medicine and Global Health, Department of Clinical Sciences Malmö, Lund University, Malmö, Sweden; Division of Occupational and Environmental Medicine, Lund University, Lund, Sweden; ZOE Limited, London, UK; Department of Twin Research and Genetic Epidemiology, King’s College London, London, UK; Lund University Diabetes Center, Department of Clinical Sciences, Skåne University Hospital, Malmö, Sweden; Harvard Chan School of Public Health, Boston, MA, USA; Department of Clinical Sciences in Malmö, Diabetic Complications Unit, Lund University Diabetes Centre, Sweden; Department of Medical Sciences, Molecular Epidemiology, and Science for Life Laboratory, Uppsala University, Sweden; Division of Occupational and Environmental Medicine, Lund University, Lund, Sweden; Clinical Studies Sweden, Forum South, Skåne University Hospital, Lund, Sweden

**Keywords:** Coronavirus, SARS-CoV-2, non-pharmaceutical interventions, policy evaluation, quasi-experimental design

## Abstract

**Background:**

This paper investigates the impact of a non-mandatory and age-specific social distancing recommendation on isolation behaviors and disease outcomes in Sweden during the first wave of the COVID-19 pandemic (March to July, 2020). The policy stated that people aged 70 years or older should avoid crowded places and contact with people outside the household.

**Methods:**

We used a regression discontinuity design—in combination with self-reported isolation data from COVID Symptom Study Sweden (n = 96,053; age range: 39-79 years) and national register data (age range: 39-100+ years) on severe COVID-19 disease (hospitalization or death, n = 21,804) and confirmed cases (n = 48,984)—to estimate the effects of the policy.

**Results:**

Our primary analyses showed a sharp drop in the weekly number of visits to crowded places (-13%) and severe COVID-19 cases (-16%) at the 70-year-threshold. These results imply that the age-specific recommendations prevented approximately 1,800 to 2,700 severe COVID-19 cases, depending on model specification.

**Conclusion:**

It seems that the non-mandatory, age-specific recommendations helped control COVID-19 disease during the first wave of the pandemic in Sweden, as opposed to not implementing a social distancing policy aimed at older adults. Our study provides empirical data on how populations may react to non-mandatory, age-specific social distancing policies in the face of a novel virus.

**Supplementary material:**

Online appendix with figures, tables, extra methods and results.

## Keywords

Coronavirus; SARS-CoV-2; non-pharmaceutical interventions; policy evaluation; quasi-experimental design

## Introduction

During the first wave of the COVID-19 pandemic in spring 2020, 19 countries in the EU/EEA and the UK implemented social distancing or 'stay-at-home' recommendations for risk groups or vulnerable populations[1]. One of these countries were Sweden, where the Public Health Agency issued a non-mandatory recommendation for individuals aged 70 or over, i.e., the most vulnerable population group concerning severe COVID-19 disease, to avoid contact with persons outside the household and crowded places (e.g., stores, public transportation)[2].

Systematic reviews of the literature on non-pharmaceutical interventions (NPIs) against COVID-19 suggest that social distancing recommendations and 'stay-at-home' orders were moderately effective at reducing the incidence of COVID-19 disease[3,4]. The tendency to comply with NPIs against COVID-19 seems to increase with age[5,6], and survey data from Sweden suggest that many followed the age-specific recommendation[7,8]. However, it remains unclear how effective the policy was in preventing COVID-19 disease among older adults in Sweden.

In this paper, we employ a regression discontinuity design[9] to isolate the additional effect of Sweden's age-specific recommendation on social distancing behaviors and disease outcomes during the first wave of the COVID-19 pandemic, beyond the effects of general recommendations that were present at the time.

## Methods

### The recommendations and context

The first wave of the COVID-19 pandemic hit Sweden between March and July, 2020. On March 16, 2020, the Public Health Agency in Sweden issued a specific recommendation that individuals aged 70 years or older should avoid crowded places and contact with people outside the household[8], which was in effect until October 2020[8] (just prior to the second wave). The same recommendation was given to individuals younger than 70 years if they had at least one of the following risk factors: high blood pressure, heart disease, lung disease, obesity, diabetes, or receiving immunosuppressant treatment.

### Social distancing outcome measures

We used data from COVID Symptom Study Sweden (CSSS)[10], an app-based study that collects data for epidemiologic surveillance and prediction of severe acute respiratory syndrome coronavirus 2 (SARS-CoV-2) infection via daily self-reports of disease symptoms[11–13]. On their first use of the app, participants self-reported their year of birth, sex, height, weight, and postal code. They also completed a health survey with questions about pre-existing health conditions. Participation was voluntary and anyone aged 18 or above living in Sweden could download the CSS app and participate after providing informed consent. The app received considerable attention in national and local press in the areas surrounding the two founding universities (Uppsala and Lund). Overall, participants were more healthy, less likely to live in disadvantaged areas, and less likely to be smokers than the general population[10].

As the first wave occurred during the spring of 2020, we considered individuals who were 70 years of age at the end of 2019 (i.e., born in 1949) to be exposed to the age-specific social distancing recommendations. From May 7 to September 29, 2020, the app also included a weekly question about the levels of isolation during the last seven days. The respondents were asked: (i) "In the last week, how many times have you visited somewhere with lots of people (e.g., groceries, public transport, work)?", (ii) "In the last week, how many times have you been outside, with little interaction with people outside your household (e.g., exercise)?", and (iii) "In the last week, how many times have you visited a healthcare provider (e.g., hospital, clinic, dentist, pharmacy)?". As described further below, our analysis focuses on individuals close to the 70-year-threshold. However, it requires data from younger and older individuals to model the relationship between these social distancing measures and age. We decided *a priori* to include period-specific averages of the social distancing measures for individuals born before 1980 (i.e., age 39 at the end of 2019) in the study, and there were too few participants born each year before 1940 (79 years) to be included in the analysis of the social distancing data. Participants also had to have at least one observation of isolation data from the period when the social distancing questions were asked up until the end of the first wave of the pandemic ([May 7 – July 31 2020]) ($n$ = 96,053). We averaged the three social distancing measures for each respondent to form a weekly average during this period. Table S1 in the Supplementary material contains an overview of characteristics of the entire sample and for individuals close to the age threshold for the recommendations (65-69 years, 70-74 years).

Due to the isolation policy's aim to reduce visits to crowded places, measure (i) was our primary measure of social distancing. Going outdoors with limited physical interaction was fine according to the recommendations. Measure (ii) should therefore not be affected. It was less clear what to expect for measure (iii). It was recommended that a courier (such as a younger relative) collect prescriptions from pharmacies. Measure (iii) did, however, include in-person healthcare visits, for which postponement could be considered an adverse effect.

### Disease outcomes

We also investigated population-level effects on severe cases (hospitalizations or deaths attributable to COVID-19). We obtained national data on all individuals born before 1980 and coded a binary indicator for whether they had at least one inpatient COVID-19 disease episode or had died due to COVID-19 disease during the first wave (March 16 – July 31, 2020; $n$ population = 5,396,837; $n$ severe cases = 21,804). The inpatient data were retrieved from the National Patient Register[14] and mortality data from the Cause of Death Register[15] (see Supplementary material for a detailed description). The retrieved data also contained information on year of birth, home address postal code, and sex.

As a secondary disease outcome, we used the number of confirmed infections by polymerase chain reaction (PCR) testing obtained from the SmiNet database at the Public Health Agency ($n$ confirmed cases = 48,984). It was mandatory for all clinical laboratories in Sweden to report PCR tests positive for SARS-CoV-2 to SmiNet during the COVID-19 pandemic[10]. Tests were highly selective during the first wave, and positive cases represented mostly people who either needed treatment or were being tested because they worked in the health care industry. Thus, absolute effects should be interpreted with caution. Nevertheless, as explained in the following section, our design compares individuals aged just above and below 70 years. Therefore, relative estimates can still be meaningful, assuming that testing probabilities were equal close to this threshold.

### Regression discontinuity analysis

We relied on a sharp regression discontinuity design (RDD) to estimate the effect of the recommendations on social distancing behaviors and disease outcomes as a discontinuous function of age, in years at the end of 2019, at the 70-year-threshold. The design has, e.g., been used to estimate the effects of early antiretroviral therapy for HIV patients[16] and other age-specific policies (e.g., minimum drinking age laws and co-payments in healthcare)[17,18].

Causal effects can be estimated in observational data without controlling for confounders by exploiting changes induced by arbitrary thresholds, such as an age limit[16]. If no other causes of the outcome changes discontinuously at the policy threshold, the RDD estimates will reflect causal effects at the threshold (i.e., for people who are precisely 70 years of age)[19]. We are not aware of any other policies that might have affected social distancing or COVID-19 disease at the 70-year-threshold. The ability to isolate may be affected by retirement, but 65 is the most common retirement age in Sweden, and retiring at 70 is rare[20]. Hence, retirement should not bias the results by causing a discontinuity at the 70-year-threshold.

Our implementation follows the RDD estimation and reporting guidelines outlined by Athey & Imbens[21], Boon et al[9], and Gelman & Imbens[22]. While our primary interest is in individuals just above and below the 70-year-threshold, RDD estimation requires fitting models to estimate the relationship between outcome variables and age. This estimation is usually performed within a small age window around the threshold (also known as *bandwidth*). The outcome-age relationship is not of primary interest but helps capture the effects of confounding variables that develop smoothly with age. However, we have to use appropriate model specification and bandwidth to avoid model misspecification bias[22]. Complex model specifications in RDD analyses are prone to overfitting, and Gelman & Imbens caution against using models with high-order polynomials (greater than linear or quadratic)[22]. We therefore used local linear and quadratic regressions to estimate the jump in the outcomes at the threshold. In each analysis, we used a data-driven bandwidth selection method to identify the mean squared error (MSE) optimal window around the 70-year-threshold[23]. The larger the bandwidth (i.e., the age window used in the analysis), the more individuals are included, which increases the precision of the effect estimates. However, the risk of model misspecification bias also increases. The data-driven procedure aims to identify the largest possible window in which the relationship between the outcome and age is approximately linear (or quadratic, depending on the model). The analyses were performed using the *rdrobust* package (version: winter 2020) for Stata (version 16.1)[24]. Futher details are provided in the Supplementary material.

#### *Subgroup analyses*

As the pandemic did not affect all regions equally, we conducted subgroup analyses by geographical area (Stockholm, which was hit particularly hard in the first wave, versus the rest of Sweden). We also stratified results by sex to investigate how the underlying risk affected the effect of the recommendation. In the social distancing data, which contained information on medical risk factors, we also considered two additional subgroups: those without any and those with at least one of the following six risk factors communicated by the Public Health Agency in May 2020: obesity (body mass index $\geq 30$), diabetes, lung disease, cancer, heart disease, or on immunosuppressant medication.

#### *Sensitivity analyses*

We performed recommended sensitivity, balance, and falsification checks to assess the risk of bias[9]. We present these analyses in the Supplementary material. In summary, analyses with alternative bandwidths are similar to the main results. The data also passed standard falsification and balance checks (e.g., no evidence of sorting or discontinuities on covariates).

### Ethics approval

The Swedish Ethical Review Authority has approved CSSS and the collection of the register data used in this study (DNR 2020-01803 with addendums 2020-04006, 2020-04145, 2020-04451, 2020-07080, and 2021-02316).

## Results

### Principal findings

The study participants went to crowded places 5.4 times a week, outdoors with limited interaction 8.8 times a week, and to healthcare providers 0.5 times a week on average during follow-up (see Table S1 for details). Figure 1 shows how these behaviors varied by age, alongside the fitted values from local linear (Figure 1A) and quadratic (Figure 1B) regressions estimated within the optimal windows around the 70-year-threshold. The analysis suggests that the policy threshold is associated with a sharp decline in the average number of times older adults visited crowded places (e.g., stores) during the first wave of the pandemic (-0.47 [95% CI: -.89, -.05] times less per week in the entire sample, which corresponds to a 13% reduction; Table 1; Figure 1). We found no evidence of discontinuities at the 70-year-threshold on being outside with little interaction or visits to healthcare providers (Table 1; Figure 1).

Figure 2 shows estimated effects on the incidence of severe COVID-19 disease and all confirmed cases per 1,000 population at the national level during the first wave of the pandemic, and Table 2 contains the effect estimates expressed as incidence rate differences and rate ratios. Overall, it appears that the recommendations may lead to a reduction in COVID-19 disease at the age threshold compared to a scenario without the age-specific recommendations (Table 2; Figure 2). The local linear estimates indicate a 16% reduction in both severe COVID-19 cases (incidence rate ratio [IRR] = 0.84 [95% CI: 0.73, 1.00]) and the number of confirmed cases (IRR = 0.84 [95% CI: 0.69, 1.08]) at the 70-year-threshold, although the confidence interval for confirmed cases overlaps the null (Table 2). For severe cases, the estimate was slightly larger in the quadratic specification (IRR = 0.78 [95% CI: 0.64, 0.99]). Our calculation in Box S1 uses these numbers to estimate the impact of the recommendations, assuming that the relative effect is the same for everyone older than 70 years. The results imply that the policy prevented 1803 [95% CI: 19, 3636] severe cases (2737 [95% CI: 87, 5388] according to the quadratic estimate) and 624 [95% CI: 7, 1257] deaths (quadratic estimate: 947 [95% CI: 30, 1864]).

### Subgroup analyses

The results from the subgroup analyses are presented in Table 1 (social distancing outcomes), Table 2 (disease outcomes), and Figures S1-S5 (RDD plots). These estimates were generally imprecise, and the observed differences between subgroups should therefore be interpreted with due caution.

Nonetheless, we found an indication that the effect on visits to crowded places was larger among individuals without risk factors than those with at least one risk factor, larger among men than women, and larger in Stockholm County than in the rest of Sweden (Table 1; Figures S1-S2). Exploring these subgroups further, we found that the effect on visits crowded places appeared to be limited to men (independent of risk group status) and women without other risk factors. We found no indication of an effect among women with other risk factors (Figure S3), but they also isolated themselves more than the other groups even at younger ages (Figure S3).

For the disease outcomes, stronger absolute effects were suggested among men than among women (Table 1; Figures S4-S5), which is consistent with the social distancing results. However, no effect on COVID-19 disease was observed for Stockholm County, where results were inconclusive (Table 2, Figures S4-S5).

## Discussion

The results suggest that Swedish 70-year-olds isolated themselves more than those just below 70 years, implying that at least parts of the population adhered to the non-mandatory, age-specific recommendations communicated by the Swedish Public Health Agency.

The results were generally in line with expectations. In particular, we found that the effect was limited to visits to crowded places, which is the social distancing outcome we assumed would be affected most by the recommendations. The impact on social distancing also seems to have caused a drop in disease outcomes at the 70-year-threshold. We were unable to draw firm conclusions from our subgroup analyses, however. Results were inconsistent and inconclusive for Stockholm County, where the pandemic hit particularly hard during the first wave in Sweden. Statistical uncertainty aside, our data suggest also that men may have experienced larger disease risk reductions from the age-specific recommendation than women, while the impact on behaviors seem to have been roughly equal. It seems reasonable that men would benefit more due to their higher disease risk. However, previous research suggests that women tend to comply with NPIs against COVID-19 to a greater extent that men[25], which we did not find evidence of for this particular policy. People with other risk factors (especially among women) also appeared to be more willing to self-isolate even at younger ages, which could—at least in part—be a consequence of the recommendations aimed at people with other risk factors.

Our study adds to the body of knowledge about the effectiveness of non-pharmaceutical interventions for the control of novel viruses. Previous evidence regarding the effectiveness of social distancing recommendations and stay-at-home orders indicate that they were moderately effective in reducing disease transmission during the COVID-19 pandemic[3,4,26], which is in line with our results. We are not aware of any other empirical studies evaluating the effects of age-specific restrictions or recommendations. Our study therefore provides new insights into how populations may react to age-specific social distancing policies. The notion of higher risks among older people most likely became widespread among the public early during the pandemic, which probably had a general effect on social behavior across age groups irrespective of recommendations. This notwithstanding, the discontinuities we observe suggest that the age-specific recommendation had an effect in addition to that general effect on from the pandemic and other policies.

A modeling study conducted by the Swedish Public Health Agency estimated that the age-specific recommendation prevented between 2,100 and 3,600 hospitalizations and 750 to 1,312 deaths during March – September 2020[8]. Their study is based on assumptions about the reduction in the number of contacts. Our study provides direct empirical support that the recommendations helped control the outbreak, with impact estimates that are slightly smaller but close to the simulation study results (Box S1).

The Swedish response to the COVID-19 pandemic was relatively lenient compared to most countries and mainly included non-mandatory recommendations to the public during the first wave of the pandemic[27]. Part of the strategy was to shield vulnerable population groups while keeping society as open as possible. The age-specific recommendation was an important aspect of this strategy, and it is conceivable that the effects are dependent not only on the acceptance among those targeted but also on which other population-level measures (such as limiting the size of gatherings and restrictions directed towards non-essential businesses) that were implemented during the same period[26]. The Swedish public also has high levels of social trust and trust in its government[28,29], which may have played a role in the success of the age-specific recommendations[30]. However, data from other countries suggest that individual psychological factors (e.g., beliefs about efficacy of the recommendations) may have a larger effect on compliance with NPIs against COVID-19 than institutional trust[31].

Our results should also be interpreted in the light of concerns about adverse effects on mental health [7,32–36]. In fact, age-specific recommendations were withdrawn in October 2020 due to these concerns[8]. Investigating potential adverse effects is therefore an important avenue for future research.

### Strengths and limitations

Our study relied on an RD design, which allows for causal effect estimation in observational data under relatively weak assumptions[9]. Other policies that use the same threshold may, however, bias the results[19]. Sweden had no other relevant policies using a 70-year threshold during the COVID-19 pandemic. The observed discontinuities were also isolated to the expected outcome variables, suggesting causality. The validity of our estimates also depends on appropriate modeling of the age-outcome relationship. We followed the current best practice recommendations, which is to fit simple models (linear or quadratic) within a data-driven bandwidth (age window) around the threshold[21,22]. A typical concern is that the conclusions may depend heavily on the selected bandwidth[9], but our results are robust to other reasonable bandwidth choices as shown in the Supplementary material. A limitation is that RDD can only be used to estimate effects for persons who are exactly 70 years old. The estimates may not generalize to older parts of the targeted age group, and the calculations in Box S1 should, therefore, be interpreted with caution. In addition, while urging older adults to isolate themselves seems to have been a better alternative than encouraging no one to isolate, our data do not permit us to explore what would have happened if the policy had been aimed at a broader age group.

A key strength of our study was the availability of detailed and complete register data for severe COVID-19 disease, which most likely limited the extent of outcome misclassification, together with repeated assessment of social distancing during the study period.

However, there are some noteworthy limitations to our social distancing data. Firstly, the social distancing measures were self-reported and could therefore be prone to bias if respondents feel pressured to provide a socially acceptable response[37,38]. While the overall levels of the isolation data may be affected, this would only be a problem for the validity of the effect estimates if persons just above 70 years falsely reported greater levels of isolation as a consequence of the policy. Secondly, participants in the app study were healthier and less disadvantaged than the general population[10]. Thus, the social distancing effect estimates may not generalize to the Swedish population if socioeconomically advantaged groups comply more with non-mandatory recommendations, as suggested by data from Norway and the US[39,40]. Reassuringly, none of these problems affect the disease outcome data, and the fact that we find an effect in both datasets suggests that our overall conclusions are valid.

Another limitation to our study is that social distancing data was only available after May 6, 2020, and thus presents the latter part of the first pandemic wave. Our study was further limited by the selective PCR testing strategy in Sweden during the spring of 2020, which meant that we could not quantify effects on infection rates in absolute terms. Moreover, since we only had access to data on year of birth and lacked data on cohabitation with persons above 70 years, our estimate may suffer from exposure misclassification bias. In both cases, we believe that the misclassification would lead to an underestimation of the true effect. Another limitation was that we could not stratify effects on disease outcomes by medical risk factors, as such register data were not available for the present study.

### Conclusion

The age-specific social distancing recommendations appear to have had an additional impact on disease risks and social distancing behaviors beyond the general recommendations that were present at the time. This suggests that non-mandatory social distancing recommendations targeting risk groups may reduce disease transmission during a pandemic, protect against severe disease, and save lives.

## Funding

CB, JB & AN were funded by a research grant from the Swedish Research Council for Health, Working life and Welfare (Forte; grant no. 2020-00962). JB had additional funding for the project from the Swedish research council (2019-00198 and 2021-04665), Sweden's innovation agency (Vinnova 2021-02648) and from Lund University (internal funding for thematic collaboration initiatives). The COVID Symptom Study Sweden (CSSS) was funded by the Swedish Heart-Lung Foundation (20190470, 20140776), Swedish Research Council (EXODIAB, 2009-1039; 2014-03529), European Commission (ERC-2015-CoG - 681742 NASCENT), and Swedish Foundation for Strategic Research (LUDC-IRC, 15-0067) to MG or PF. TF was a holder of a European Research Council Starting Grant (801965). JG was funded by a postdoctoral research grant from the Swedish Civil Contingencies Agency (2019-08944). None of the funding entities had any role in study design, data analysis, data interpretation, or the writing of this manuscript.

## Competing interests

COVID Symptom Study Sweden (CSSS) is a strictly non-commercial research project. PF consults for and has stock options in ZOE Limited relating to the PREDICT nutrition studies, which are entirely separate from the COVID Symptom Study app development and COVID-19 research. JCP works for ZOE Limited. The remaining authors have no competing interests to declare.

## Acknowledgments

We are grateful to the participants of the COVID Symptom Study Sweden for their data contribution. We would also like to thank all the researchers involved in the global COVID Symptom Study consortium, in particular Andrew T. Chan (Clinical and Translational Epidemiology Unit, Massachusetts General Hospital and Harvard Medical School), Sébastien Ourselin (School of Biomedical Engineering and Imaging Sciences, King's College London), Tim D Spector (Department of Twin Research and Genetic Epidemiology, King's College London), and Jonathan Wolf (ZOE Limited), for their parts in the design of questionnaires and data collection. We also thank Martin

Adiels and colleagues for compiling and sharing summary data on the share of severe cases that occurred in care homes at our request.

## Data availability

The data supporting this article constitute sensitive personal information and can only be made available to researchers with an approval from the Swedish Ethical Review Authority. Please contact the corresponding author for further details. The analysis code can be found in the Supplementary appendix.

## Key points

- The Swedish Public Health Agency issued a non-mandatory recommendation for older adults (70+ years) to avoid crowded places and contact with people outside the household during the first wave of the COVID-19 pandemic.
- Using a regression discontinuity design, we find a sharp drop in the number of weekly visits to crowded places at the 70-year-threshold.
- We also find a drop in severe COVID-19 cases at the same age, indicating that the policy prevented approximately 1,800 to 2,700 cases compared to having no special recommendations for older adults.
- Despite being non-mandatory, the age-specific recommendations seemed to have had an impact on the spread of disease, which implies that social distancing recommendations targeting risk groups may reduce disease transmission during a pandemic, protect against severe disease, and save lives.

## Tables

**Table 1.** Regression discontinuity estimates of the effect of the social distancing recommendations for people aged 70+ years in Sweden on the level of isolation during the first wave of the COVID-19 pandemic, by type of activity, model specification and subgroup.

| Group | Model specification: Local linear | | Model specification: Local quadratic | |
|---|---|---|---|---|
| | Additive effect | Relative effect | Additive effect | Relative effect |
| *i. Weekly visits to crowded places (e.g., stores, public transportation)* | | | | |
| Full sample | -.47 (-.89, -.05) | .87 (.77, .98) | -.57 (-1.25, .11) | .84 (.71, 1.04) |
| ***Subgroup*** | | | | |
| No risk factors | -.57 (-1.12, -.02) | .84 (.73, .99) | -.73 (-1.54, .09) | .81 (.67, 1.03) |
| At least one risk factor | -.32 (-.95, .31) | .90 (.76, 1.12) | -.56 (-1.50, .37) | .84 (.66, 1.14) |
| Men | -.56 (-1.14, .02) | .86 (.74, 1.01) | -.72 (-1.47, .04) | .82 (.69, 1.01) |
| Women | -.35 (-.91, .22) | .89 (.75, 1.09) | -.58 (-1.45, .28) | .83 (.66, 1.11) |
| Stockholm County | -.83 (-1.74, .08) | .79 (.64, 1.03) | -.72 (-1.74, .31) | .81 (.63, 1.11) |
| Rest of Sweden | -.38 (-.83, .08) | .89 (.79, 1.03) | -.60 (-1.37, .17) | .84 (.69, 1.06) |
| *ii. Weekly number of times gone outside with limited interaction* | | | | |
| Full sample | -.50 (-1.22, .21) | .95 (.89, 1.02) | -.16 (-1.21, .89) | .98 (.89, 1.10) |
| ***Subgroup*** | | | | |
| No risk factors | -.62 (-1.52, .29) | .94 (.87, 1.03) | .21 (-1.22, 1.64) | 1.02 (.89, 1.2) |
| At least one risk factor | -.41 (-1.68, .86) | .96 (.85, 1.10) | -.40 (-1.79, .98) | .96 (.84, 1.11) |
| Men | -.85 (-2.05, .35) | .92 (.82, 1.04) | -1.02 (-2.39, .35) | .90 (.80, 1.04) |
| Women | -.42 (-1.45, .62) | .96 (.87, 1.07) | 1.05 (-.58, 2.69) | 1.12 (.94, 1.38) |
| Stockholm County | -1.51 (-3.74, .71) | .87 (.73, 1.08) | -1.34 (-3.71, 1.02) | .88 (.73, 1.11) |
| Rest of Sweden | -.24 (-1.03, .55) | .98 (.9, 1.06) | -.04 (-1.11, 1.03) | 1.00 (.90, 1.12) |
| *iii. Weekly visits to healthcare provider(s)* | | | | |
| Full sample | .01 (-.02, .05) | 1.05 (.92, 1.21) | .01 (-.06, .07) | 1.03 (.83, 1.35) |
| ***Subgroup*** | | | | |
| No risk factors | .01 (-.04, .06) | 1.04 (.86, 1.30) | .01 (-.07, .08) | 1.02 (.78, 1.49) |
| At least one risk factor | .02 (-.03, .08) | 1.07 (.90, 1.30) | .03 (-.04, .10) | 1.10 (.88, 1.47) |
| Men | -.02 (-.07, .02) | .91 (.79, 1.09) | -.03 (-.08, .02) | .90 (.77, 1.09) |
| Women | .05 (-.01, .10) | 1.20 (.98, 1.53) | .02 (-.05, .10) | 1.08 (.86, 1.47) |
| Stockholm County | .03 (-.04, .10) | 1.13 (.89, 1.53) | .05 (-.06, .15) | 1.18 (.83, 2.04) |
| Rest of Sweden | .01 (-.04, .05) | 1.02 (.89, 1.21) | .00 (-.06, .06) | 1.00 (.83, 1.27) |

*Notes*: Additive estimates reflect bias-corrected effects on the difference scale (where 0 = null effect) estimated within MSE-optimal bandwidths, with 95% Eicker-Huber-White heterscedasticity-robust confidence intervals from the *rdrobust* package for Stata in parentheses. Relative estimates reflect ratios (where 1 = null effect) computed using the additive estimates (see Supplementary material for details).

**Table 2.** Regression discontinuity estimates of the effect of the social distancing recommendations for people aged 70+ years in Sweden on severe COVID-19 disease and confirmed cases during the first wave of the COVID-19 pandemic, by model specification and subgroup.

| Group | Model specification | | | |
|---|---|---|---|---|
| | Local linear | | Local quadratic | |
| | IRD (Absolute effect) | IRR (Relative effect) | IRD (Absolute effect) | IRR (Relative effect) |
| *i. Severe cases (hospitalized or dead) per 1,000 population* | | | | |
| Full sample | -.65 (-1.29, -.01) | .84 (.73, 1.00) | -.99 (-1.94, -.03) | .78 (.64, .99) |
| ***Subgroup*** | | | | |
| Men | -.84 (-1.73, .05) | .84 (.73, 1.01) | -1.47 (-2.86, -.08) | .76 (.61, .98) |
| Women | .03 (-.58, .64) | 1.01 (.80, 1.38) | -.32 (-1.32, .67) | .88 (.64, 1.40) |
| Stockholm County | .52 (-1.02, 2.06) | 1.08 (.87, 1.40) | -.44 (-2.74, 1.86) | .943 (.72, 1.35) |
| Rest of Sweden | -.86 (-1.52, -.21) | .76 (.64, .93) | -1.24 (-2.20, -.29) | .69 (.55, .90) |
| *ii. Confirmed cases per 1,000 population* | | | | |
| Full sample | -.81 (-1.93, .31) | .84 (.69, 1.08) | -.73 (-2.02, .56) | .86 (.68, 1.15) |
| ***Subgroup*** | | | | |
| Men | -.67 (-1.62, .28) | .89 (.77, 1.05) | -1.46 (-3.11, .20) | .79 (.64, 1.04) |
| Women | -.44 (-1.73, .85) | .88 (.66, 1.35) | -.43 (-1.97, 1.12) | .89 (.63, 1.52) |
| Stockholm County | .83 (-.86, 2.52) | 1.12 (.90, 1.46) | -.29 (-3.34, 2.76) | .96 (.70, 1.53) |
| Rest of Sweden | -.78 (-2.02, .46) | .83 (.65, 1.14) | -.28 (-1.86, 1.29) | .91 (.60, 1.84) |

*Notes*: Estimates reflect bias-corrected incidence rate differences per 1,000 population (IRD, i.e., absolute effects where 0 = null effect) and incidence rate ratios (IRR) (i.e., relative effects where 1 = null effect) estimated within MSE-optimal bandwidths, with 95% Eicker-Huber-White heterscedasticity-robust confidence intervals from the *rdrobust* package for Stata in parentheses.

## Figure legends

**Figure 1.** Regression discontinuity plots for the impact of Sweden's age-specific isolation recommendations on social distancing behaviors at the 70-year-threshold with binned means (gray dots) and fitted values (black lines) from local linear (panel A) and quadratic (panel B) regressions estimated within mean-squared-error-optimal bandwidths around the threshold, for three social distancing measures: (i) mean weekly visits to crowded places, (ii) mean weekly outdoor episodes with no or limited interaction, and (iii) mean weekly visits to healthcare providers.

**Figure 2.** Regression discontinuity plots for the impact of Sweden's age-specific isolation recommendations on COVID-19 disease incidence per 1,000 population at the 70-year-threshold with binned means (gray dots) and fitted values (black lines) from local linear (panel A) and quadratic (panel B) regressions estimated within the mean-squared-error-optimal bandwidths around the threshold, for two disease outcome measure: (i) severe cases (hospitalized or dead), and (ii) all confirmed cases. The y-axis is plotted on a logarithmic scale to enable better visualization of the data close to the 70-year-threshold.

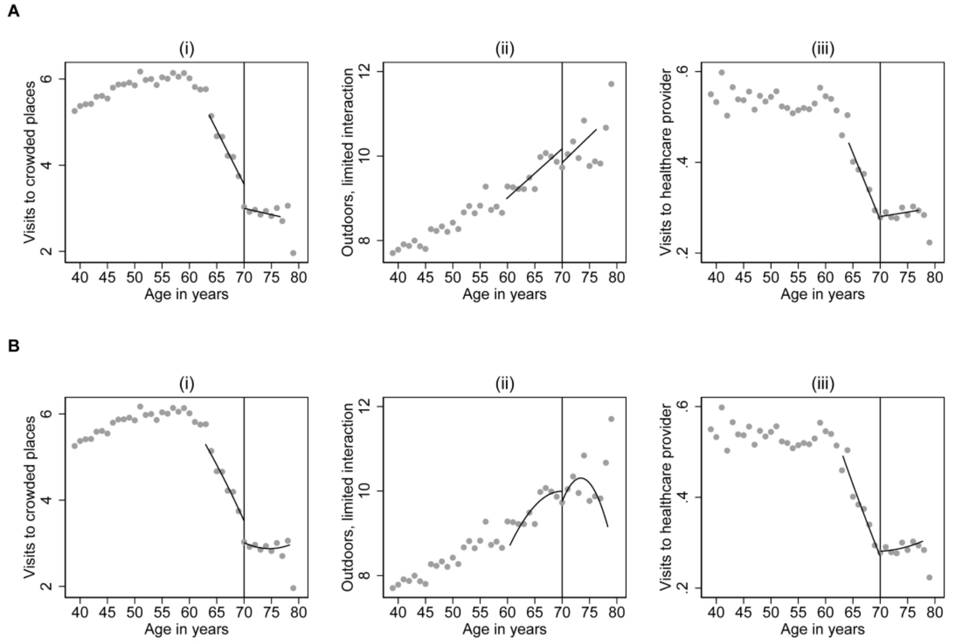

Figure 1. Regression discontinuity plots for the impact of Sweden's age-specific isolation recommendations on social distancing behaviors at the 70-year-threshold with binned means (gray dots) and fitted values (black lines) from local linear (panel A) and quadratic (panel B) regressions estimated within mean-squared-error-optimal bandwidths around the threshold, for three social distancing measures: (i) mean weekly visits to crowded places, (ii) mean weekly outdoor episodes with no or limited interaction, and (iii) mean weekly visits to healthcare providers.

127x88mm (1200 x 1200 DPI)

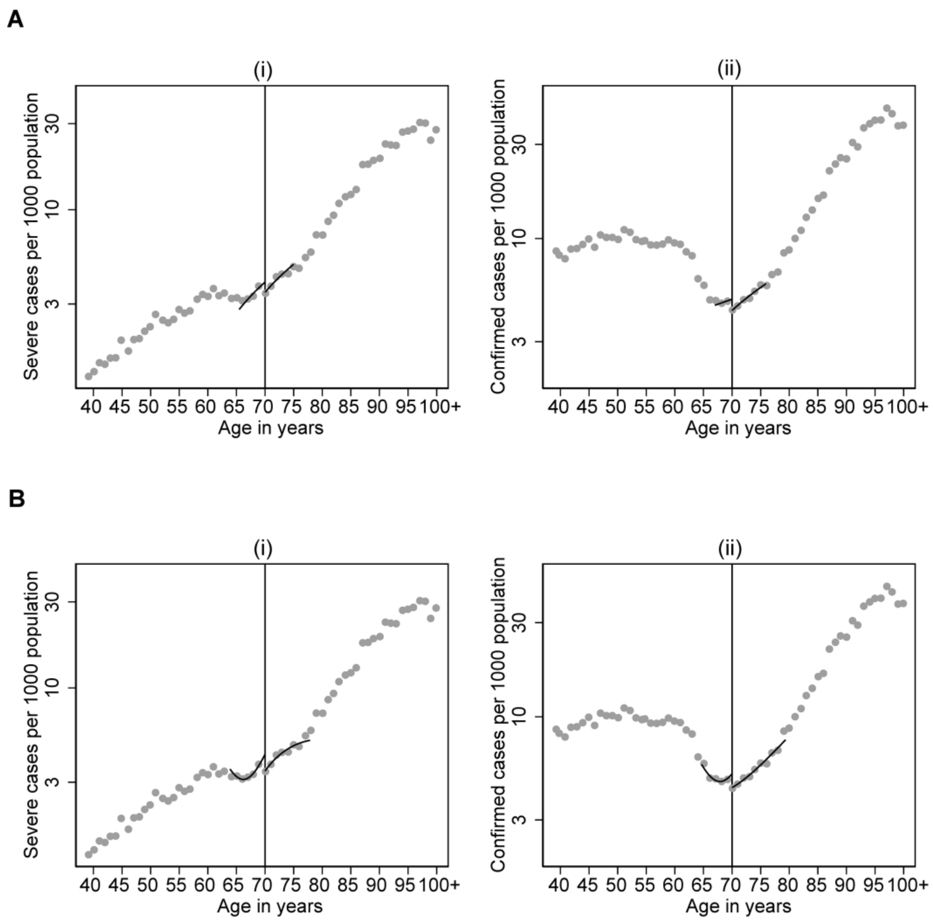

Figure 2. Regression discontinuity plots for the impact of Sweden's age-specific isolation recommendations on COVID-19 disease incidence per 1,000 population at the 70-year-threshold with binned means (gray dots) and fitted values (black lines) from local linear (panel A) and quadratic (panel B) regressions estimated within the mean-squared-error-optimal bandwidths around the threshold, for two disease outcome measure: (i) severe cases (hospitalized or dead), and (ii) all confirmed cases. The y-axis is plotted on a logarithmic scale to enable better visualization of the data close to the 70-year-threshold.

63x63mm (1200 x 1200 DPI)

## Supplementary Material

ckac101_Supplementary_Data

