# Supplementary material for: A regression discontinuity analysis of the social distancing recommendations for older adults in Sweden during COVID-19
Source: Eur J Public Health. 2022 Aug 13;32(5):799–806. doi: 10.1093/eurpub/ckac101 (PMC9384721; doi:10.1093/eurpub/ckac101)
Supplement: ckac101_Supplementary_Data [file ckac101_supplementary_data.docx]

Supplementary material

for

“A regression discontinuity analysis of the social distancing recommendations for older adults in Sweden during COVID-19”

by

Carl Bonander^1*^, Debora Stranges^2^, Johanna Gustavsson^3^, Matilda Almgren^4^, Malin Inghammar^5^, Mahnaz Moghaddassi^6^, Anton Nilsson^2^, Paul W Franks^7,8^, Maria Gomez^9^, Tove Fall^10^, Jonas Björk^2,4^ and COVID Symptom Study Sweden

^1 Health Economics & Policy, School of Public Health & Community Medicine, Sahlgrenska Academy, University of Gothenburg, Sweden^

^2 Division of Occupational and Environmental Medicine, Lund University, Lund, Sweden^

^3 Centre for Societal Risk Research, Karlstad University, Sweden^

^4 Clinical Studies Sweden, Forum South, Skåne University Hospital, Lund, Sweden^

^5 Department of Clinical Sciences Lund, Section for Infection Medicine, Skåne University Hospital, Lund University, Lund, Sweden^

^6 Social Medicine and Global Health, Department of Clinical Sciences Malmö, Lund University, Malmö, Sweden^

^7 Lund University Diabetes Center, Department of Clinical Sciences, Skåne University Hospital, Malmö, Sweden^

^8 Harvard Chan School of Public Health, Boston, MA, USA^

^9 Department of Clinical Sciences in Malmö, Diabetic Complications Unit, Lund University Diabetes Centre, Sweden^

^10 Department of Medical Sciences, Molecular Epidemiology, and Science for Life Laboratory, Uppsala University, Sweden^

***Corresponding author. Address:** Carl Bonander, School of Public Health and Community Medicine, Institute of Medicine, Sahlgrenska Academy, University of Gothenburg,
SE-405 30 Gothenburg, Sweden. E-mail: [carl.bonander@gu.se](mailto:carl.bonander@gu.se)

[Supplementary methods 3](#_Toc100732589)

[Classification of severe COVID-19 cases 3](#_Toc100732590)

[Detailed description of the estimation strategy 3](#_Toc100732591)

[Method for calculating relative effects from RD estimates 4](#_Toc100732592)

[Supplementary results 6](#_Toc100732593)

[Sensitivity analyses 6](#_Toc100732594)

[Impact estimates 7](#_Toc100732595)

[Supplementary tables 8](#_Toc100732596)

[Supplementary figures 15](#_Toc100732597)

[Software code 22](#_Toc100732598)

[Code for Table S1 22](#_Toc100732599)

[Regression discontinuity analysis (example) 25](#_Toc100732600)

[Density test 27](#_Toc100732601)

[References 29](#_Toc100732602)

# Supplementary methods

## Classification of severe COVID-19 cases

We extracted our primary disease outcome data from the Swedish National Patient Register^1^ and the Cause of Death Register^2^, both of which cover the entire Swedish population. Our primary endpoint was hospitalization (an inpatient episode) or death due to COVID-19 during the period March 16, 2020 to July 31, 2020. Classification was made according to the International Classification of Diseases and Related Health Problems, tenth revision (ICD-10). The inpatient data were retrieved from the National Patient Register, using the emergency ICD-10 codes U07.1 (COVID-19, confirmed by laboratory testing) and U07.2 (COVID-19, virus not identified). Mortality data were retrieved from the Cause of Death Register, where COVID-19 deaths are defined using ICD-10 codes U07.1, U07.2, and B34.2 (Coronavirus infection, unspecified). Persons diagnosed with U07.1 and/or U07.2 within 30 days of their date of death are also included in the definition of COVID-19 deaths, regardless of the cause(s) listed in the cause-of-death certificate. The databases were linked at the individual level by the National Board of Health and Welfare using personal identification numbers (PIN). The final dataset was cleaned of PINs and pseudonymized to before being sent to us.

## Detailed description of the estimation strategy

We used a data-driven estimation strategy to determine the mean-squared-error (MSE) optimal bandwidth to include in each analysis. Specifically, we followed the approach suggested by Calonico, Cattaneo & Titiunik,^3^ which determines an MSE-optimal window for each analysis depending on the model specification and data. Their estimator also includes a bias-correction to account for bias owing to the bias-variance trade-off in the MSE-optimal bandwidth, where the bias is estimated and accounted for by fitting a polynomial regression of one degree higher than the main model (e.g., a quadratic bias model for estimates based on local linear regression). This procedure allows for valid and robust statistical inference with data-driven bandwidth selection.^3^ As recommended, we used a triangular kernel to linearly down-weight observations away from the policy threshold.^4^ We also allowed the slopes and optimal bandwidth lengths to differ on either side of the threshold. We based our inferences on Eicker-Huber-White (EHW) heteroscedasticity-robust standard errors, as Kolesár & Rothe^5^ recently showed that the EHW method exhibits better coverage rates for discrete running variables, compared to the older recommendation to cluster the running variable,^6^ in data similar to ours. The analyses were performed using the *rdrobust* package (version: winter 2020) for Stata (version 16.1).^7^

## Method for calculating relative effects from RD estimates

The *rdrobust* package provides estimates of the additive effect on the outcome. Using the potential outcomes framework, the estimated quantity can be written as

(1) $\beta=E\left[ Y_{i}\left( 1 \right) | Age_{i}=70 \right]-E\left[ Y_{i}\left( 0 \right) | Age_{i}=70 \right],$

where $Y_{i}\left( 1 \right)$ and $Y_{i}\left( 0 \right)$ are the potential outcomes if individual *i* is exposed and not exposed to the recommendations, respectively. A relative (ratio) version of this estimate can be written as:

(2) ${E\left[ Y_{i}\left( 1 \right) | Age_{i}=70 \right]}/{E\left[ Y_{i}\left( 0 \right) | Age_{i}=70 \right]}$

The conditional average of the realized outcomes $Y_{i}$ among individuals aged exactly 70 years provides a direct estimate of$E\left[ Y_{i}\left( 1 \right) | Age_{i}=70 \right]$ because all individuals aged 70 years are exposed to the recommendations, but $E\left[ Y_{i}\left( 0 \right) | Age_{i}=70 \right]$ needs to be inferred. Re-arranging Equation (1) yields the following expression:

(3) $E\left[ Y_{i}\left( 0 \right) | Age_{i}=70 \right]=E\left[ Y_{i}\left( 1 \right) | Age_{i}=70 \right]-\beta$

To estimate relative effects, we can therefore use

(4) $\hat{E}\left[ Y_{i} | Age_{i}=70 \right]/(\hat{E}\left[ Y_{i} | Age_{i}=70 \right]-\hat{\beta})$,

where $\hat{E}\left[ Y_{i} | Age_{i}=70 \right]$ is the average outcome among individuals aged exactly 70 years, and $\hat{\beta}$ is the additive effect estimate from the regression discontinuity analysis. To get 95% confidence intervals, we replace $\hat{\beta}$ with the lower and upper bounds of the confidence intervals for $\hat{\beta}$.

# Supplementary results

## Sensitivity analyses

In this section, we present the results from a broad range of sensitivity and falsification checks typical for RD designs.^8^ Specifically, we (1) checked for discontinuities in the other covariates presented in **Table S1** to assess violations of the continuity assumption, (2) conducted covariate-adjusted regression discontinuity analyses to adjust for any jumps in covariates at the age threshold, (3) varied the age window by ±1 and ±2 years from the MSE-optimal bandwidths to assess sensitivity to stochastic errors in the determination of the optimal bandwidth, and (4) checked for sorting in the number of observations just above and below the threshold in the social distancing data to rule out effects of the recommendations on selection into the study sample using the *rddensity* package for Stata.^9,10^

For the social distancing outcomes, varying the bandwidth by ±1 and ±2 years around the MSE-optimal age window gave rise to similar estimates as in the main analysis for visits to crowded places (**Tables S2-S3**). Estimates of the effect on the other two social distancing outcomes changed sign in a few analyses and subgroups but were generally consistent with the main analyses in that they do not provide any strong indications of an effect (**Tables S2-S3**). The covariate falsification checks did not show any meaningful and robust jumps in covariates (**Table S4)**, and covariate-adjusted estimates were very similar to the main analyses (**Table S4**). Finally, we found no evidence of sorting of observations around the 70-year-threshold (**Figure S6**; p-value for a jump in the density of observations at the threshold: 0.95).

The disease outcome results appeared slightly more sensitive to bandwidth selection than the social distancing estimates (especially to increasing the age window), which was not surprising given that the disease outcomes are highly non-linear in windows greater than the MSE-optimal bandwidths in most groups (as can be seen in **Figure 2 in the main text**). However, all estimates were of the same sign as the main estimates in each alternative bandwidth, except for the effect estimate for severe disease outcomes among women, which was close to zero in the main analysis and changed sign from negative to positive with some of the alternative bandwidths (**Tables S5-S6**).

## Impact estimates

**Box S1. Approximate calculation of the total impact of the age-specific recommendations on COVID-19 disease in the age group 70+ years during the first pandemic wave in Sweden.**

We here estimate the total impact of the recommendations on severe COVID-19 disease and deaths, assuming that the relative effect is constant for all ages above 70 years. During the study period, 21,804 cases of severe COVID-19 disease occurred in Sweden according to our definition (hospitalized or dead); 12,258 (56%) of these cases were aged 70+ years, 46% of whom died due to the disease. An ongoing study that links COVID-19 case data with data on living conditions indicate that 22.2% of severe cases in this age group occurred in care homes (personal communication, Martin Adiels, University of Gothenburg, 2021-06-22). It is unlikely that the recommendations could have prevented these cases. Combining these numbers with ours suggest that approximately 12258*(1-0.222)=9537 severe cases occurred in the community-dwelling population. Dividing this number by the relative local linear estimate (IRR = 0.841 [95% CI: 0.724, 0.998]) implies that 11340 severe cases would have occurred without recommendations. This suggests that the policy prevented 1803 [95% CI: 19, 3636] severe cases during the first wave (2737 [95% CI: 87, 5388] according to the quadratic estimate). Turning to the number of deaths, the external data show that a larger share of deaths (41.5%) than severe non-fatal cases (7.26%) occurred in care homes. Repeating the above calculations for the number of deaths in our data (n = 5,639), our policy effect estimates imply that 624 [95% CI: 7, 1257] deaths were prevented (quadratic estimate: 947 [95% CI: 30, 1864]).

# Supplementary tables

**List of Supplementary tables**

[**Table S1.** Characteristics of the sample born 1940 - 1980 and average social distancing behaviors stratified by risk group. 9](#_Toc80687166)

[**Table S2.** Estimates of the policy effect on social distancing outcomes from local linear sensitivity analyses varying the bandwidth (BW; i.e., age window) by ±1 and ±2 years from the data-driven MSE-optimal BW. A subtraction indicates a smaller BW (using observations closer to the threshold), whereas an addition gives a larger BW (using more observations farther away from the threshold). 10](#_Toc80687167)

[**Table S3.** Estimates of the policy effect on social distancing outcomes from quadratic linear sensitivity analyses varying the bandwidth (BW; age window) by ±1 and ±2 years from the data-driven MSE-optimal BW. A subtraction indicates a smaller BW (using observations closer to the threshold), whereas an addition gives a larger BW (using more observations farther away from the threshold). 11](#_Toc80687168)

[**Table S4.** Regression discontinuity estimates for jumps in covariates at the policy threshold using local linear and local quadratic estimation with data-driven (MSE-optimal) bandwidth selection, and covariate-adjusted estimates for the social distancing outcomes. 12](#_Toc80687169)

[**Table S5.** Estimates of the policy effect on disease outcomes from local linear sensitivity analyses varying the bandwidth (BW; age window) by ±1 and ±2 years from the data-driven MSE-optimal BW. A subtraction indicates a smaller BW (using observations closer to the threshold), whereas an addition gives a larger BW (using more observations farther away from the threshold). 13](#_Toc80687170)

[**Table S6.** Estimates of the policy effect on disease outcomes from local quadratic sensitivity analyses varying the bandwidth (BW; age window) by ±1 and ±2 years from the data-driven MSE-optimal BW. A subtraction indicates a smaller BW (using observations closer to the threshold), whereas an addition gives a larger BW (using more observations farther away from the threshold). 14](#_Toc80687171)

**Table S1.** Characteristics of the sample born 1940 - 1980 and average social distancing behaviors stratified by risk group.

| Characteristic | *Total* | *65-69 yrs* | *70-74 yrs* |
| --- | --- | --- | --- |
|  | N=96,053 | N=9,358 | N=5,992 |
| ***Demographics*** |  |  |  |
| Age at the end of 2019 - mean (SD) | 54.5 (10.2) | 66.9 (1.4) | 71.4 (1.1) |
| Women - % | 60.2% | 55.7% | 50.5% |
| Lives in Stockholm County - % | 22.9% | 19.9% | 19.5% |
| ***Risk factors*** |  |  |  |
| Obese (body mass index ≥ 30) - % | 19.4% | 16.2% | 13.7% |
| Diabetes - % | 4.6% | 8.8% | 8.6% |
| Lung disease - % | 13.8% | 13.5% | 12.7% |
| Cancer - % | 1.4% | 3.0% | 3.6% |
| Heart disease - % | 6.4% | 12.2% | 15.2% |
| Takes immunosuppressants - % | 4.7% | 5.8% | 6.0% |
| Has at least one risk factor - % | 38.0% | 42.5% | 42.0% |
| ***Level of isolation, by type of activity*** |  |  |  |
| Visited crowded places, n times (weekly) - mean (SD) | 5.4 (6.3) | 4.3 (6.6) | 2.9 (5.2) |
| Visited crowded places, n times (weekly) - median (IQR) | 4.0 (2.3-6.3) | 2.8 (1.6-4.8) | 1.9 (0.8-3.3) |
| Outdoors with limited interaction, n times (weekly) - mean (SD) | 8.8 (9.6) | 9.8 (11.6) | 10.0 (12.0) |
| Outdoors with limited interaction, n times (weekly) - median (IQR) | 6.3 (4.0-10.0) | 6.6 (4.2-10.5) | 6.5 (4.0-11.0) |
| Visited healthcare provider, n times (weekly) - mean (SD) | 0.5 (1.1) | 0.4 (0.7) | 0.3 (0.4) |
| Visited healthcare provider, n times (weekly) - median (IQR) | 0.2 (0.0-0.6) | 0.2 (0.0-0.5) | 0.1 (0.0-0.4) |

*^Notes:^* ^The level of isolation reflects weekly average of available self-reports between the period 2020-05-07 and 2020-07-31.^

**Table S2.** Estimates of the policy effect on social distancing outcomes from local linear sensitivity analyses varying the bandwidth (BW; i.e., age window) by ±1 and ±2 years from the data-driven MSE-optimal BW. A subtraction indicates a smaller BW (using observations closer to the threshold), whereas an addition gives a larger BW (using more observations farther away from the threshold).

| **Group** | **Bandwidth (BW)** | | | | | All of same sign? |
| --- | --- | --- | --- | --- | --- | --- |
|  | -2 years | -1 years | MSE-optimal | +1 years | +2 years |  |
| *i. Weekly visits to crowded places* | | | | | | |
| All | -.48 (-.98, .03) | -.49 (-.95, -.03) | -.47 (-.89, -.05) | -.41 (-.80, -.01) | -.33 (-.70, .05) | Yes |
| Risk+ | -.33 (-1.08, .42) | -.35 (-1.03, .32) | -.32 (-.95, .31) | -.24 (-.83, .35) | -.14 (-.71, .43) | Yes |
| Risk- | -.6 (-1.26, .05) | -.61 (-1.21, -.02) | -.57 (-1.12, -.02) | -.49 (-1.01, .03) | -.44 (-.93, .05) | Yes |
| Men | -.57 (-1.25, .11) | -.60 (-1.23, .02) | -.56 (-1.14, .02) | -.50 (-1.05, .06) | -.46 (-.99, .07) | Yes |
| Women | -.39 (-1.06, .28) | -.39 (-1.00, .22) | -.35 (-.91, .22) | -.26 (-.79, .27) | -.17 (-.68, .33) | Yes |
| Stockholm | -.84 (-1.88, .20) | -.84 (-1.81, .12) | -.83 (-1.74, .08) | -.80 (-1.66, .07) | -.79 (-1.62, .03) | Yes |
| Rest of Sweden | -.45 (-.99, .09) | -.44 (-.92, .05) | -.38 (-.83, .08) | -.28 (-.71, .15) | -.19 (-.60, .21) | Yes |
| *ii. Number of times gone outside with limited interaction* | | | | | | |
| All | -.44 (-1.22, .35) | -.5 (-1.25, .25) | -.5 (-1.22, .21) | -.49 (-1.18, .2) | -.47 (-1.14, .2) | Yes |
| Risk+ | -.41 (-1.87, 1.05) | -.41 (-1.76, .94) | -.41 (-1.68, .86) | -.38 (-1.59, .83) | -.37 (-1.52, .78) | Yes |
| Risk- | -.51 (-1.48, .47) | -.57 (-1.51, .37) | -.62 (-1.52, .29) | -.63 (-1.51, .24) | -.62 (-1.47, .23) | Yes |
| Men | -.92 (-2.38, .54) | -.95 (-2.25, .36) | -.85 (-2.05, .35) | -.78 (-1.91, .34) | -.71 (-1.77, .35) | Yes |
| Women | -.20 (-1.32, .92) | -.35 (-1.42, .73) | -.42 (-1.45, .62) | -.47 (-1.47, .53) | -.47 (-1.44, .5) | Yes |
| Stockholm | -1.05 (-3.76, 1.65) | -1.35 (-3.77, 1.08) | -1.51 (-3.74, .71) | -1.60 (-3.67, .47) | -1.63 (-3.58, .31) | Yes |
| Rest of Sweden | -.18 (-1.04, .68) | -.23 (-1.05, .60) | -.24 (-1.03, .55) | -.21 (-.98, .55) | -.21 (-.95, .53) | Yes |
| *iii. Weekly visits to healthcare provider(s)* | | | | | | |
| All | .03 (-.02, .08) | .02 (-.02, .06) | .01 (-.02, .05) | .02 (-.02, .06) | .02 (-.01, .06) | Yes |
| Risk+ | .05 (-.02, .13) | .03 (-.03, .1) | .02 (-.03, .08) | .03 (-.03, .09) | .03 (-.04, .09) | Yes |
| Risk- | .01 (-.05, .07) | .01 (-.04, .06) | .01 (-.04, .06) | .02 (-.03, .07) | .03 (-.01, .07) | Yes |
| Men | -.03 (-.08, .02) | -.03 (-.07, .02) | -.02 (-.07, .02) | -.02 (-.06, .03) | -.01 (-.05, .04) | Yes |
| Women | .07 (-.01, .15) | .05 (-.01, .12) | .05 (-.01, .1) | .05 (0.0, .10) | .07 (.01, .12) | Yes |
| Stockholm | .05 (-.04, .13) | .05 (-.03, .13) | .03 (-.04, .1) | .04 (-.03, .10) | .04 (-.02, .1) | Yes |
| Rest of Sweden | .02 (-.03, .08) | .01 (-.03, .06) | .01 (-.04, .05) | .02 (-.03, .06) | .02 (-.02, .06) | Yes |

*^Notes:^* ^Estimates reflect bias-corrected absolute effects (where 0 = null effect), with 95% robust confidence intervals from the^ *^rdrobust^* ^package for Stata in paranthases. Risk+ = at least one medical risk factor (see main text), Risk- = no medical risk factors other than old age.^

**Table S3.** Estimates of the policy effect on social distancing outcomes from quadratic linear sensitivity analyses varying the bandwidth (BW; age window) by ±1 and ±2 years from the data-driven MSE-optimal BW. A subtraction indicates a smaller BW (using observations closer to the threshold), whereas an addition gives a larger BW (using more observations farther away from the threshold).

| **Group** | **Bandwidth (BW)** | | | | | All of same sign? |
| --- | --- | --- | --- | --- | --- | --- |
|  | -2 years | -1 years | MSE-optimal | +1 years | +2 years |  |
| *i. Weekly visits to crowded places* | | | | | | |
| All | -.36 (-1.27, .55) | -.43 (-1.19, .33) | -.57 (-1.25, .11) | -.70 (-1.32, -.08) | -.69 (-1.27, -.12) | Yes |
| Risk+ | -.21 (-1.41, .98) | -.38 (-1.42, .66) | -.56 (-1.5, .37) | -.64 (-1.5, .23) | -.57 (-1.36, .23) | Yes |
| Risk- | -.56 (-1.53, .41) | -.67 (-1.55, .22) | -.73 (-1.54, .09) | -.74 (-1.5, .02) | -.75 (-1.46, -.04) | Yes |
| Men | -.86 (-1.72, -.01) | -.78 (-1.58, .01) | -.72 (-1.47, .04) | -.64 (-1.36, .08) | -.52 (-1.21, .16) | Yes |
| Women | -.47 (-1.71, .78) | -.59 (-1.66, .49) | -.58 (-1.45, .28) | -.89 (-1.78, .01) | -.85 (-1.68, -.02) | Yes |
| Stockholm | -.85 (-1.97, .26) | -.80 (-1.87, .27) | -.72 (-1.74, .31) | -.66 (-1.65, .33) | -.68 (-1.63, .28) | Yes |
| Rest of Sweden | -.45 (-1.54, .63) | -.53 (-1.41, .35) | -.60 (-1.37, .17) | -.71 (-1.4, -.01) | -.70 (-1.34, -.06) | Yes |
| *ii. Number of times gone outside with limited interaction* | | | | | | |
| All | .06 (-1.16, 1.28) | -.06 (-1.19, 1.07) | -.16 (-1.21, .89) | -.26 (-1.25, .74) | -.32 (-1.26, .62) | No |
| Risk+ | -.37 (-1.86, 1.13) | -.37 (-1.81, 1.06) | -.40 (-1.79, .98) | -.51 (-1.85, .82) | -.62 (-1.91, .67) | Yes |
| Risk- | .67 (-1.05, 2.39) | .40 (-1.16, 1.95) | .21 (-1.22, 1.64) | .00 (-1.34, 1.34) | -.19 (-1.45, 1.07) | No |
| Men | -1.17 (-2.71, .38) | -1.12 (-2.57, .32) | -1.02 (-2.39, .35) | -.93 (-2.24, .37) | -.88 (-2.12, .36) | Yes |
| Women | 1.78 (-.27, 3.84) | 1.43 (-.37, 3.24) | 1.05 (-.58, 2.69) | .75 (-.77, 2.27) | .48 (-.95, 1.91) | Yes |
| Stockholm | -1.32 (-3.95, 1.31) | -1.27 (-3.75, 1.22) | -1.34 (-3.71, 1.02) | -1.45 (-3.72, .81) | -1.70 (-3.87, .48) | Yes |
| Rest of Sweden | .07 (-1.14, 1.28) | .04 (-1.09, 1.17) | -.04 (-1.11, 1.03) | -.10 (-1.11, .92) | -.11 (-1.08, .86) | No |
| *iii. Weekly visits to healthcare provider(s)* | | | | | | |
| All | .05 (-.03, .13) | .04 (-.03, .11) | .01 (-.06, .07) | .01 (-.04, .06) | .01 (-.04, .06) | Yes |
| Risk+ | .03 (-.04, .10) | .03 (-.04, .10) | .03 (-.04, .10) | .03 (-.05, .10) | .02 (-.05, .10) | Yes |
| Risk- | .03 (-.07, .14) | .03 (-.05, .12) | .01 (-.07, .08) | .00 (-.07, .06) | -.02 (-.08, .05) | No |
| Men | -.04 (-.09, .01) | -.03 (-.09, .02) | -.03 (-.08, .02) | -.02 (-.07, .03) | -.01 (-.06, .04) | Yes |
| Women | .05 (-.04, .13) | .01 (-.07, .10) | .02 (-.05, .10) | .04 (-.03, .11) | .05 (-.02, .11) | Yes |
| Stockholm | .04 (-.1, .17) | .07 (-.05, .19) | .05 (-.06, .15) | .03 (-.06, .13) | .02 (-.07, .11) | Yes |
| Rest of Sweden | .03 (-.04, .11) | .00 (-.07, .07) | .00 (-.06, .06) | .00 (-.05, .06) | .00 (-.05, .05) | Yes |

*^Notes:^* ^Estimates reflect bias-corrected absolute effects (where 0 = null effect), with 95% robust confidence intervals from the^ *^rdrobust^* ^package for Stata in paranthases. Risk+ = at least one medical risk factor (see main text), Risk- = no medical risk factors other than old age.^

**Table S4.** Regression discontinuity estimates for jumps in covariates at the policy threshold using local linear and local quadratic estimation with data-driven (MSE-optimal) bandwidth selection, and covariate-adjusted estimates for the social distancing outcomes.

| Balance check | Local linear | Local quadratic |
| --- | --- | --- |
| *A. Tests for discontinuities in covariates at threshold* | | |
| Women - % | -1.39 (-4.41, 1.62) | -2.86 (-7.27, 1.54) |
| Lives in Stockholm - % | 1.52 (-.88, 3.92) | 1.37 (-1.91, 4.65) |
| Obese (body mass index >= 30) - % | 1.69 (-.68, 4.07) | 2.78 (.08, 5.48) |
| Diabetes - % | -1.89 (-3.53, -.26) | -1.35 (-3.41, .71) |
| Lung disease - % | -1.45 (-3.38, .47) | -1.15 (-3.64, 1.34) |
| Cancer - % | .52 (-.84, 1.88) | 1.23 (-.47, 2.94) |
| Heart disease - % | -.56 (-2.76, 1.65) | -.28 (-2.72, 2.16) |
| Takes immunosuppressants - % | -.40 (-1.80, 1.00) | -1.9 (-4.23, .44) |
| *B. Covariate-adjusted estimates for discontinuity in social distancing outcomes* | | |
| Visits to crowded places | -.50 (-.92, -.07) | -.57 (-1.26, .12) |
| Outside with little interaction | -.50 (-1.20, .21) | -.15 (-1.20, .90) |
| Visits to healthcare provider | .02 (-.02, .05) | .01 (-.05, .07) |

*^Notes:^* ^Estimates reflect bias-corrected absolute effects (where 0 = null effect) estimated within MSE-optimal bandwidths, with 95% robust confidence intervals from the^ *^rdrobust^* ^package for Stata in parentheses. We could only perform covariate-adjusted estimation in the social distancing data, since our disease dataset did not contain covariates.^

**Table S5.** Estimates of the policy effect on disease outcomes from local linear sensitivity analyses varying the bandwidth (BW; age window) by ±1 and ±2 years from the data-driven MSE-optimal BW. A subtraction indicates a smaller BW (using observations closer to the threshold), whereas an addition gives a larger BW (using more observations farther away from the threshold).

| **Group** | **Bandwidth (BW)** | | | | | All of same sign? |
| --- | --- | --- | --- | --- | --- | --- |
|  | -2 years | -1 years | MSE-optimal | +1 years | +2 years |  |
| *i. Severe cases (dead or hospitalized) per 1,000 population* | | | | | | |
| All | -.91 (-1.85, .03) | -.77 (-1.52, -.03) | -.65 (-1.29, -.01) | -.36 (-.89, .18) | -.51 (-1.09, .07) | Yes |
| Men | -1.25 (-2.42, -.08) | -1.11 (-2.15, -.06) | -.84 (-1.73, .05) | -.65 (-1.49, .19) | -.81 (-1.7, .08) | Yes |
| Women | -.29 (-1.08, .51) | -.18 (-.89, .54) | .03 (-.58, .64) | .11 (-.48, .69) | -.01 (-.63, .61) | No |
| Stockholm | .11 (-1.61, 1.83) | .35 (-1.27, 1.96) | .52 (-1.02, 2.06) | .78 (-.69, 2.26) | .92 (-.5, 2.34) | Yes |
| Rest of Sweden | -1.09 (-2.04, -.14) | -.97 (-1.72, -.21) | -.86 (-1.52, -.21) | -.74 (-1.32, -.15) | -.56 (-1.1, -.02) | Yes |
| *ii. Confirmed cases per 1,000 population* | | | | | | |
| All | NA* | -.34 (-.87, .20) | -.81 (-1.93, .31) | -.60 (-1.28, .08) | -.66 (-1.44, .12) | Yes |
| Men | -1.11 (-2.37, .15) | -1.03 (-2.17, .10) | -.67 (-1.62, .28) | -.46 (-1.38, .46) | -.69 (-1.66, .29) | Yes |
| Women | -.19 (-.86, .47) | -.58 (-1.87, .71) | -.44 (-1.73, .85) | -.25 (-1.08, .57) | -.40 (-1.34, .54) | Yes |
| Stockholm | .40 (-1.47, 2.27) | .63 (-1.13, 2.38) | .83 (-.86, 2.52) | 1.29 (-.30, 2.89) | 1.65 (.11, 3.19) | Yes |
| Rest of Sweden | NA* | -.48 (-1.04, .07) | -.78 (-2.02, .46) | -.87 (-1.69, -.05) | -.85 (-1.55, -.14) | Yes |

*^Notes:^* ^Estimates reflect bias-corrected absolute effects (where 0 = null effect), with 95% robust confidence intervals from the^ *^rdrobust^* ^package for Stata in paranthases. *Insufficient observations to run a local regression (MSE-optimal bandwidth too close to threshold).^

**Table S6.** Estimates of the policy effect on disease outcomes from local quadratic sensitivity analyses varying the bandwidth (BW; age window) by ±1 and ±2 years from the data-driven MSE-optimal BW. A subtraction indicates a smaller BW (using observations closer to the threshold), whereas an addition gives a larger BW (using more observations farther away from the threshold).

| **Group** | **Bandwidth (BW)** | | | | | All of same sign? |
| --- | --- | --- | --- | --- | --- | --- |
|  | -2 years | -1 years | MSE-optimal | +1 years | +2 years |  |
| *i. Severe cases (dead or hospitalized) per 1,000 population* | | | | | | |
| All | -1.06 (-2.42, .3) | -1.05 (-2.16, .06) | -.99 (-1.94, -.03) | -.94 (-1.78, -.09) | -.89 (-1.66, -.12) | Yes |
| Men | -1.07 (-3.29, 1.16) | -1.33 (-3.14, .49) | -1.47 (-2.86, -.08) | -1.28 (-2.68, .13) | -1.23 (-2.52, .05) | Yes |
| Women | -.71 (-2.17, .76) | -.66 (-1.87, .56) | -.32 (-1.32, .67) | -.52 (-1.48, .43) | -.52 (-1.40, .36) | Yes |
| Stockholm | -1.14 (-3.95, 1.67) | -.80 (-3.32, 1.72) | -.44 (-2.74, 1.86) | -.68 (-2.83, 1.48) | -.47 (-2.51, 1.56) | Yes |
| Rest of Sweden | -1.24 (-2.66, .17) | -1.20 (-2.33, -.06) | -1.24 (-2.2, -.29) | -1.20 (-2.06, -.34) | -1.12 (-1.90, -.34) | Yes |
| *ii. Confirmed cases per 1,000 population* | | | | | | |
| All | -.85 (-2.85, 1.15) | -.78 (-2.24, .68) | -.73 (-2.02, .56) | -1.11 (-2.16, -.07) | -1.32 (-2.27, -.38) | Yes |
| Men | -.59 (-3.32, 2.15) | -.72 (-2.9, 1.46) | -1.46 (-3.11, .20) | -.94 (-2.57, .70) | -1.14 (-2.62, .34) | Yes |
| Women | -.82 (-3.42, 1.78) | -.97 (-2.83, .89) | -.43 (-1.97, 1.12) | -1.33 (-2.66, .00) | -1.62 (-2.82, -.41) | Yes |
| Stockholm | -2.23 (-6.2, 1.74) | -1.64 (-4.88, 1.59) | -.29 (-3.34, 2.76) | -.62 (-3.16, 1.92) | -.72 (-3.06, 1.62) | Yes |
| Rest of Sweden | -.43 (-2.51, 1.64) | -.60 (-2.09, .89) | -.81 (-2.13, .51) | -1.37 (-2.45, -.3) | -1.70 (-2.67, -.73) | Yes |

*^Notes:^* ^Estimates reflect bias-corrected absolute effects (where 0 = null effect), with 95% robust confidence intervals from the^ *^rdrobust^* ^package for Stata in paranthases. *Insufficient observations to run a local regression (MSE-optimal bandwidth too close to threshold).^

# Supplementary figures

**List of Supplementary figures**

[**Figure S1.** Regression discontinuity plots for the impact of the age-specific isolation recommendations on social distancing behaviors at the 70-year-threshold with local linear estimates in mean-squared-error-optimal bandwidths around the threshold for subgroups based on medical risk factors, sex and area (Stockholm county, rest of Sweden), for three social distancing measures: A) average weekly visits to crowded places, B) average weekly outdoor episodes with no or limited interaction, and C) average weekly visits to healthcare providers. 16](#_Toc80092113)

[**Figure S2.** Regression discontinuity plots for the impact of the age-specific isolation recommendations on social distancing behaviors at the 70-year-threshold with local quadratic estimates in mean-squared-error-optimal bandwidths around the threshold for subgroups based on medical risk factors, sex and area (Stockholm county, rest of Sweden), for three social distancing measures: A) average weekly visits to crowded places, B) average weekly outdoor episodes with no or limited interaction, and C) average weekly visits to healthcare providers. 17](#_Toc80092114)

[**Figure S3**. Regression discontinuity plots for visits to crowded places in detailed subgroups by risk factor status and sex. 18](#_Toc80092115)

[**Figure S4.** Regression discontinuity plots for the impact of the age-specific isolation recommendations on COVID-19 disease incidence per 1,000 population at the 70-year-threshold with local linear estimates in mean-squared-error-optimal bandwidths around the threshold in subgroups by sex and area (Stockholm county, rest of Sweden). A) Severe cases (hospitalized or dead), B) all confirmed cases. The incidence is presented on a logarithm scale to enable better visualization of the regions around the 70-year-threshold. 19](#_Toc80092116)

[**Figure S5.** Regression discontinuity plots for the impact of the age-specific isolation recommendations on COVID-19 disease incidence per 1,000 population at the 70-year-threshold with local quadratic estimates in mean-squared-error-optimal bandwidths around the threshold in subgroups by sex and area (Stockholm county, rest of Sweden). A) Severe cases (hospitalized or dead), B) all confirmed cases. The incidence is presented on a logarithm scale to enable better visualization of the regions around the 70-year-threshold. 20](#_Toc80092117)

[**Figure S6.** Histogram of the forcing variable age showing the frequency of observations to check for evidence of sorting of observations around the 70-year-threshold (policy threshold is indicated with a vertical line). The figure does not show evidence of sorting, as the number of observations develops smoothly across the threshold. 21](#_Toc80092118)


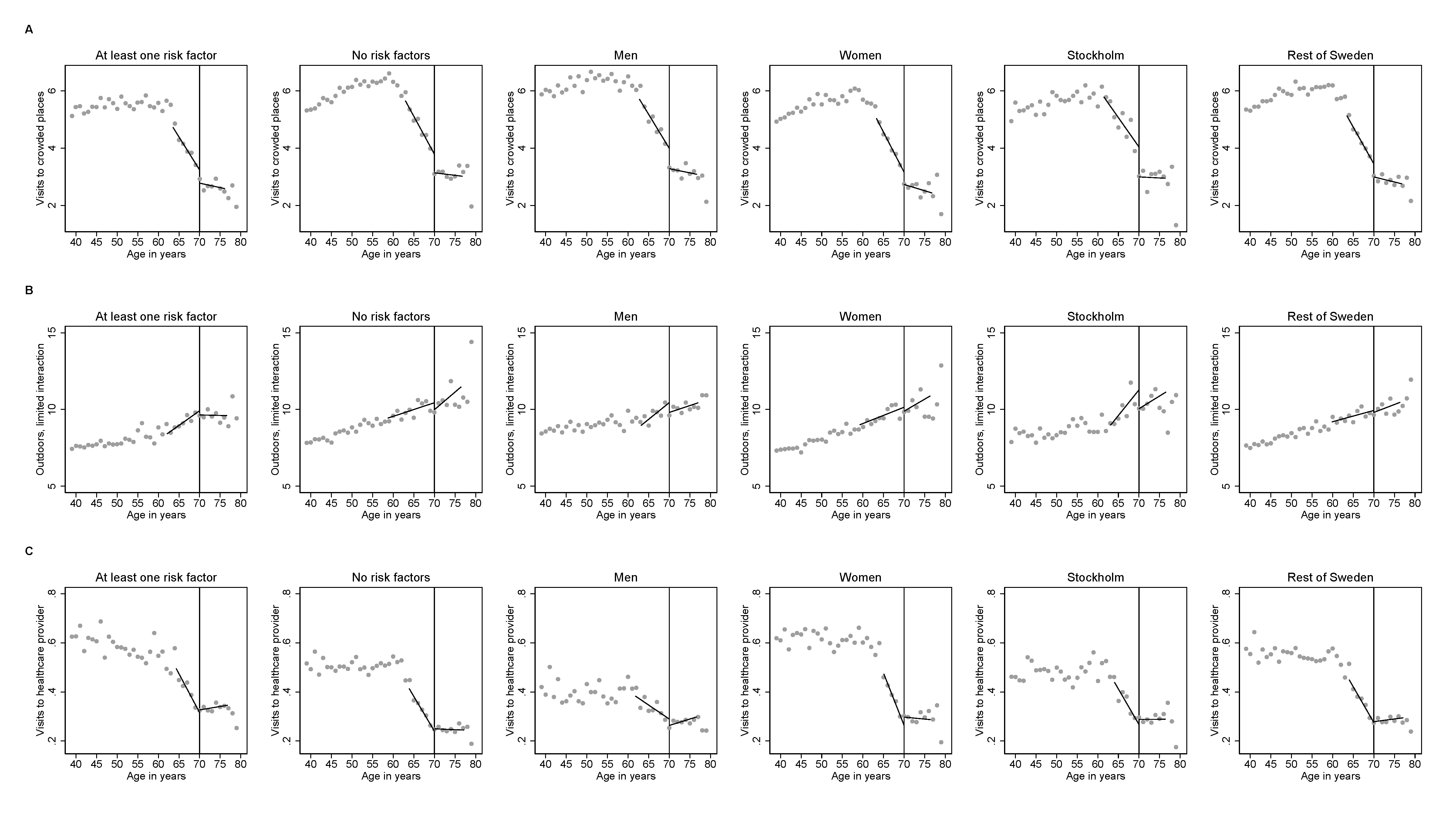


**Figure S1.** Regression discontinuity plots for the impact of the age-specific isolation recommendations on social distancing behaviors at the 70-year-threshold with local linear estimates in mean-squared-error-optimal bandwidths around the threshold for subgroups based on medical risk factors, sex and area (Stockholm county, rest of Sweden), for three social distancing measures: A) average weekly visits to crowded places, B) average weekly outdoor episodes with no or limited interaction, and C) average weekly visits to healthcare providers.


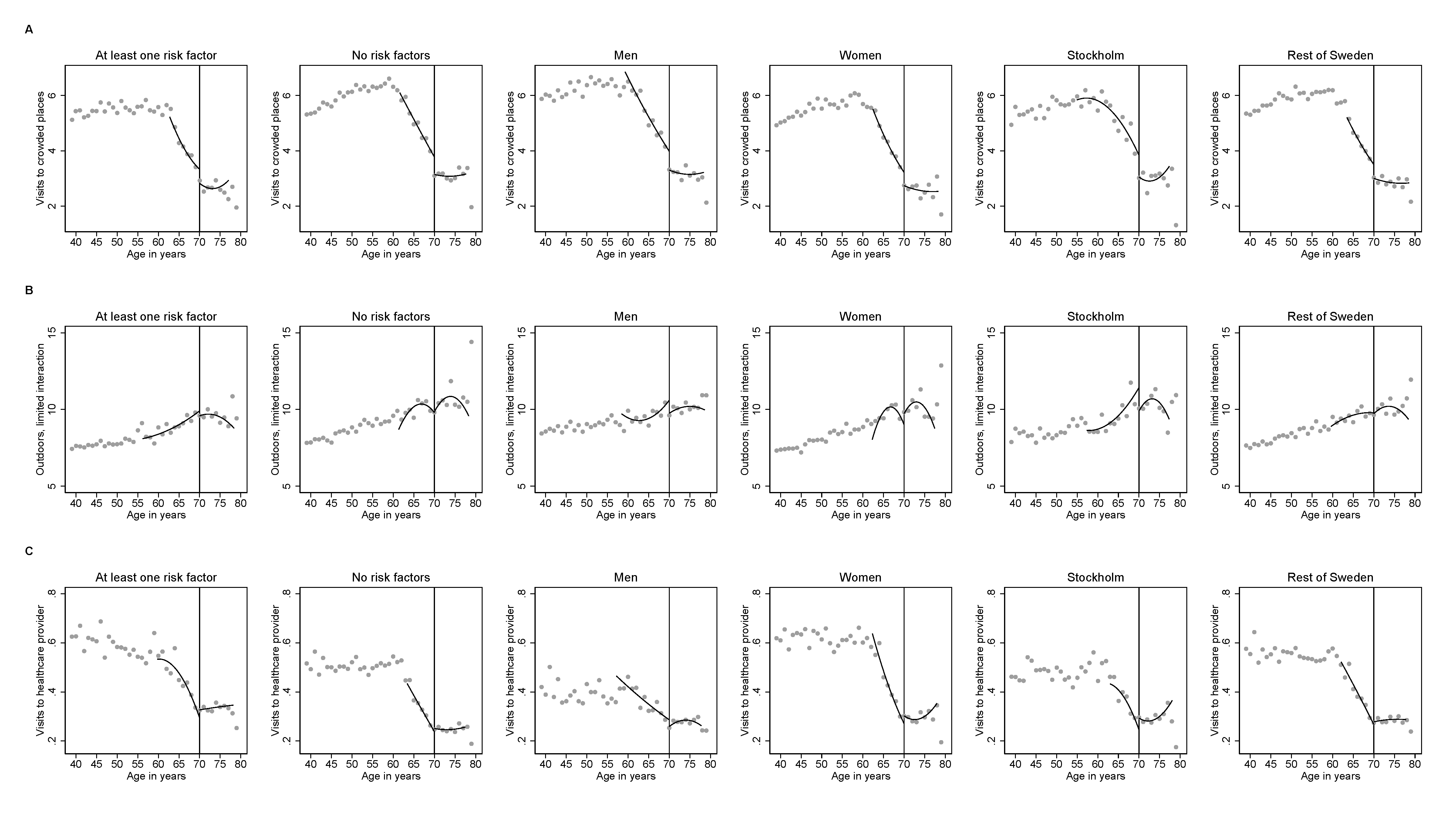


**Figure S2.** Regression discontinuity plots for the impact of the age-specific isolation recommendations on social distancing behaviors at the 70-year-threshold with local quadratic estimates in mean-squared-error-optimal bandwidths around the threshold for subgroups based on medical risk factors, sex and area (Stockholm county, rest of Sweden), for three social distancing measures: A) average weekly visits to crowded places, B) average weekly outdoor episodes with no or limited interaction, and C) average weekly visits to healthcare providers.

*
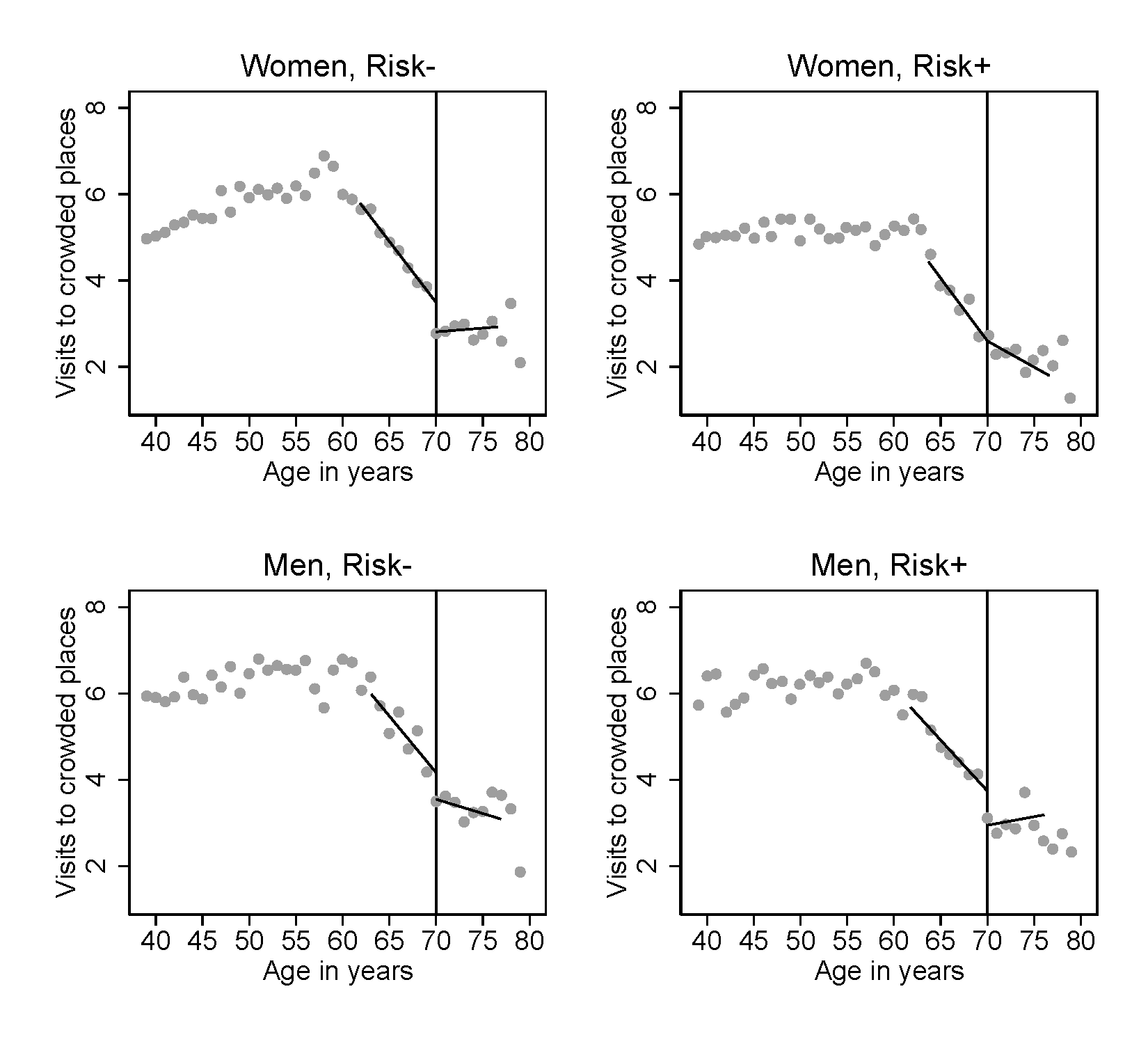
*

**Figure S3**. Regression discontinuity plots for visits to crowded places in detailed subgroups by risk factor status and sex.


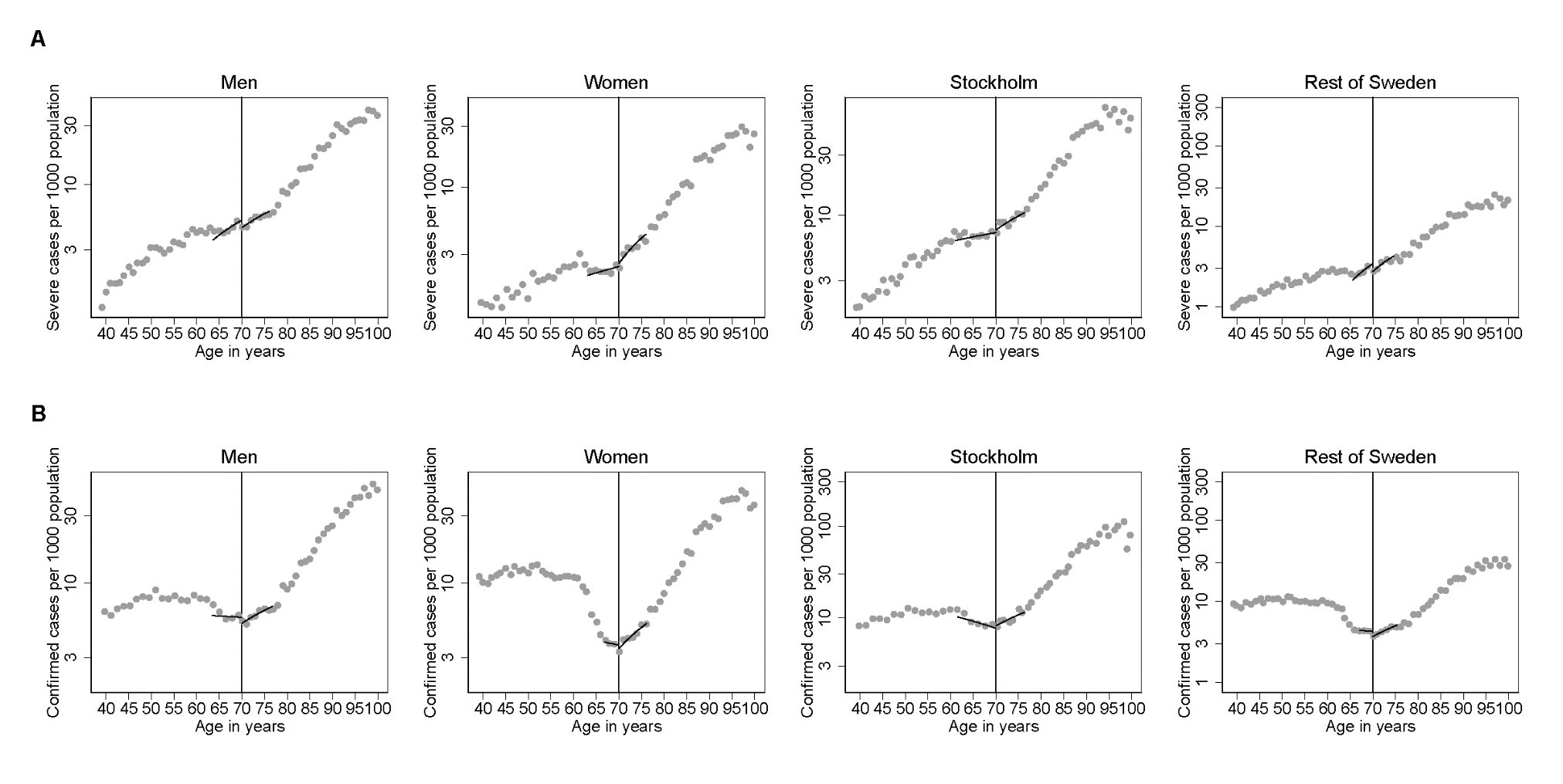


**Figure S4.** Regression discontinuity plots for the impact of the age-specific isolation recommendations on COVID-19 disease incidence per 1,000 population at the 70-year-threshold with local linear estimates in mean-squared-error-optimal bandwidths around the threshold in subgroups by sex and area (Stockholm county, rest of Sweden). A) Severe cases (hospitalized or dead), B) all confirmed cases. The incidence is presented on a logarithm scale to enable better visualization of the regions around the 70-year-threshold.


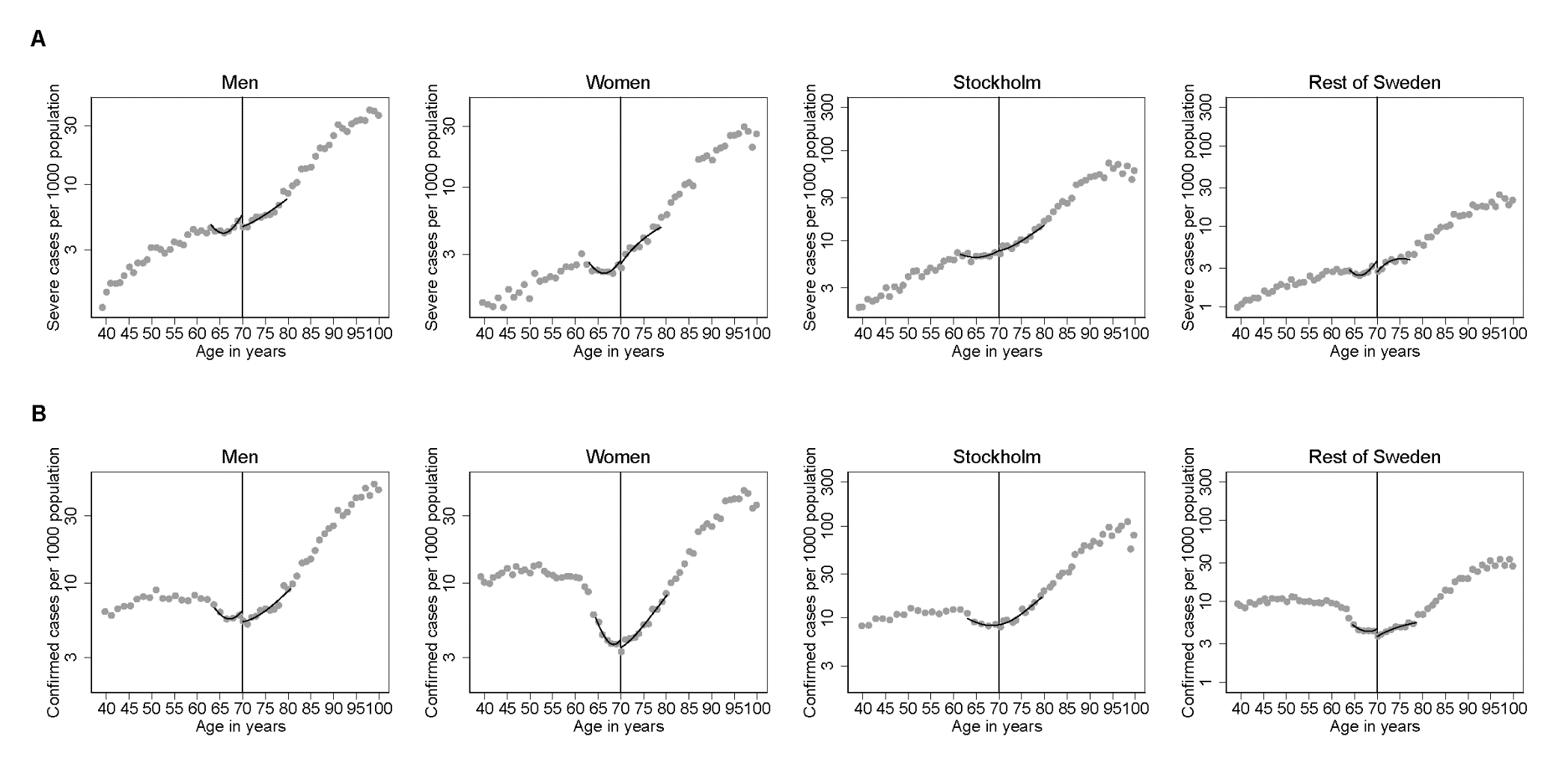


**Figure S5.** Regression discontinuity plots for the impact of the age-specific isolation recommendations on COVID-19 disease incidence per 1,000 population at the 70-year-threshold with local quadratic estimates in mean-squared-error-optimal bandwidths around the threshold in subgroups by sex and area (Stockholm county, rest of Sweden). A) Severe cases (hospitalized or dead), B) all confirmed cases. The incidence is presented on a logarithm scale to enable better visualization of the regions around the 70-year-threshold.


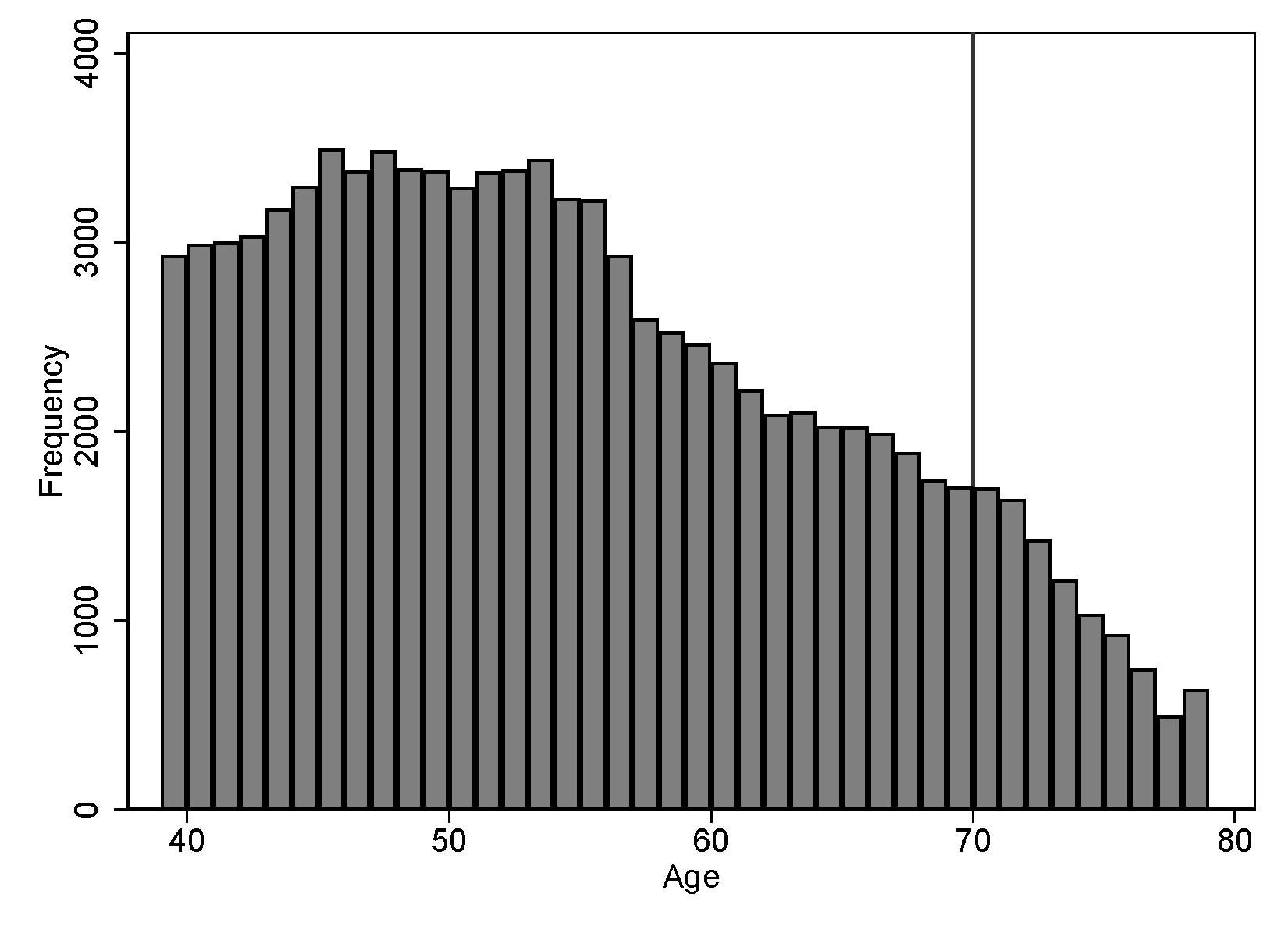


**Figure S6.** Histogram of the forcing variable age showing the frequency of observations to check for evidence of sorting of observations around the 70-year-threshold (policy threshold is indicated with a vertical line). The figure does not show evidence of sorting, as the number of observations develops smoothly across the threshold.

# Software code

In this section, we present example code and unmodified Stata output from the analyses that support the conclusions of this paper. We used Stata 16.1 SE to conduct the analyses. Besides base functions, our code relies on the *rdrobust*, *rddensity* and *table1_mc* packages, which can be installed from the web by entering the following command into Stata’s command prompt:

ssc install rdrobust

net install rddensity, from(https://raw.githubusercontent.com/rdpackages/rddensity/master/stata)

ssc install table1_mc

## Code for Table S1

// Load the data and prepare frames for output storage

clear

frame reset

cd "C:\..."

import delimited "isolation_df_before_aug.csv"

frame create restable

set scheme s1mono

frame restable: set obs 21

// 1. Prepare and label variables

gen any_lung = risk3 == 1 | risk5 == 1 | risk6 == 1

gen women = gender==0

label variable age_in_2019 "Age - mean (SD)"

label variable women "Women - %"

label variable risk1 "Obese (body mass index >= 30) - %"

label variable risk2 "Diabetes - %"

label variable any_lung "Lung disease - %"

label variable risk4 "Cancer - %"

label variable risk7 "Heart disease - %"

label variable risk9 "Takes immonusuppressants - %"

label variable risk_any "Has at least one risk factor - %"

label variable in_sthlm "Lives in Stockholm - %"

label variable isolation_lots_of_people "Visits to crowded places, n times (weekly) - mean (SD)"

label variable isolation_little_interaction "Went outdoors with limited interaction, n times (weekly) - mean (SD)"

label variable isolation_healthcare_provider "Visited healthcare provider, n times (weekly) - mean (SD)"

// 2. Create table 1 for the full sample

table1_mc, ///

vars( ///

age contn %5.1f \ ///

women bin %5.1f \ ///

in_sthlm bin %5.1f \ ///

risk1 bin %5.1f \ ///

risk2 bin %5.1f \ ///

any_lung bin %5.1f \ ///

risk4 bin %5.1f \ ///

risk7 bin %5.1f \ ///

risk9 bin %5.1f \ ///

risk_any bin %5.1f \ ///

isolation_lots_of_people contn %5.1f \ ///

isolation_lots_of_people conts %5.1f \ ///

isolation_little_interaction contn %5.1f \ ///

isolation_little_interaction conts %5.1f \ ///

isolation_healthcare_provider contn %5.1f \ ///

isolation_healthcare_provider conts %5.1f \ ///

) ///

nospace percent onecol missing total(before) ///

saving("table 1.xlsx", replace)


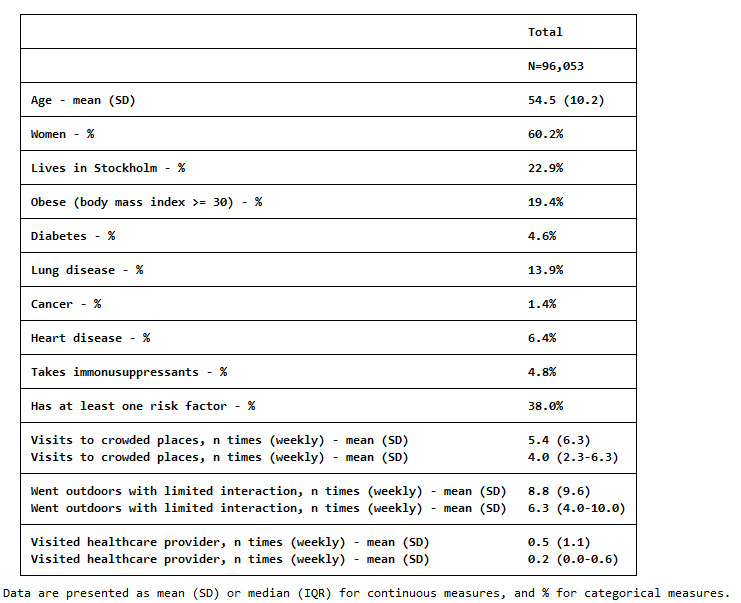


// Alternative version with age windows closer to the threshold

gen grp_age = "65-69 years" if age_in_2019>=65 & age_in_2019<70

replace grp_age = "70-74 years" if age_in_2019>=70 & age_in_2019<74

table1_mc, by(grp_age) ///

vars( ///

age contn %5.1f \ ///

women bin %5.1f \ ///

in_sthlm bin %5.1f \ ///

risk1 bin %5.1f \ ///

risk2 bin %5.1f \ ///

any_lung bin %5.1f \ ///

risk4 bin %5.1f \ ///

risk7 bin %5.1f \ ///

risk9 bin %5.1f \ ///

risk_any bin %5.1f \ ///

isolation_lots_of_people contn %5.1f \ ///

isolation_lots_of_people conts %5.1f \ ///

isolation_little_interaction contn %5.1f \ ///

isolation_little_interaction conts %5.1f \ ///

isolation_healthcare_provider contn %5.1f \ ///

isolation_healthcare_provider conts %5.1f \ ///

) ///

nospace percent onecol missing total(before) ///

saving("table 1 - age_spec.xlsx", replace)


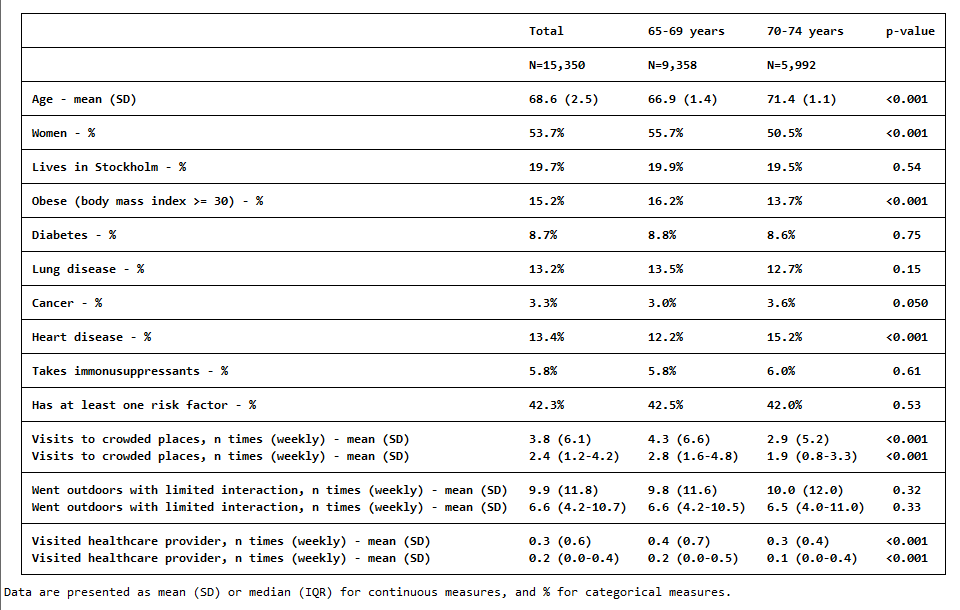


## Regression discontinuity analysis (example)

/// *** Outcome variable: isolation_lots_of_people (= “visits to crowded places”)

// 1. Linear specification using the rdrobust package (p = 1):

rdrobust isolation_lots_of_people age, c(70) p(1) kernel(triangular) bwselect(msetwo) all vce(hc0)

// Note: c(70) = 70 is the cutoff age ; kernel(triangular) = use a triangular kernel to downweight observations far from the threshold; bwselect(msetwo) = allow different lengths at either side of the threshold; all = produce bias-corrected estimates; vce(hc0) = use Eicker-Huber-White (EHW) heteroscedasticity-robust standard errors as recommended by Kolésar and Rothe for discrete running variables. Output:


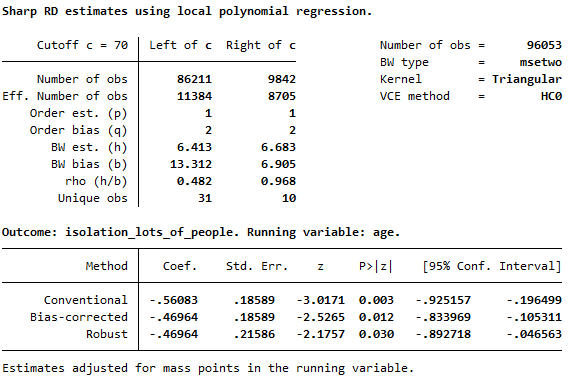


// 2. Store optimal bandwidth (age window) for plotting later

local Window_l = e(h_l)

local Window_r = e(h_r)

local b_l = e(b_l)

local b_r = e(b_r)

// 3. Store the robust estimate & CI from model 1 for the RD output table in the paper

frame restable: gen var = "Lots of people" in 1

frame restable: gen group = "All" in 1

frame restable: gen linear = string(round(`e(tau_bc)',0.01)) + " (" + string(round(`e(tau_bc)'-`e(se_tau_rb)'*1.96,0.01)) + ", " + string(round(`e(tau_bc)'+`e(se_tau_rb)'*1.96,0.01)) + ")" in 1

// Use stored estimates to calculate relative effect and store that as well

tempvar logout

gen `logout' = isolation_lots_of_people

qui sum `logout' if age==70

local ty = r(mean)

local rel_point = `ty' / (`ty' - `e(tau_bc)')

local rel_lower = `ty' / (`ty' - (`e(tau_bc)'-`e(se_tau_rb)'*1.96))

local rel_upper = `ty' / (`ty' - (`e(tau_bc)'+`e(se_tau_rb)'*1.96))

frame restable: gen linear_rel = string(round(`rel_point',0.01)) + " (" + string(round(`rel_lower',0.01)) + ", " + string(round(`rel_upper',0.01)) + ")" in 1

// This is what the frame “restable” looks like after this (it is later filled out with more estimates):


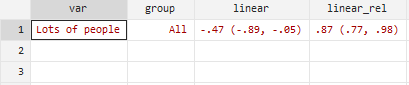


// 4. Produce the rd plot for model 1

rdplot isolation_lots_of_people age, c(70) h(`Window_l' `Window_r') p(1) kernel(triangular) graph_options(ylabel(#3) xlabel(#20) saving(g_lots_all.gph, replace) xtitle("Age in years") ytitle("Visits to crowded places") title("(i)", size(4)) legend(off))


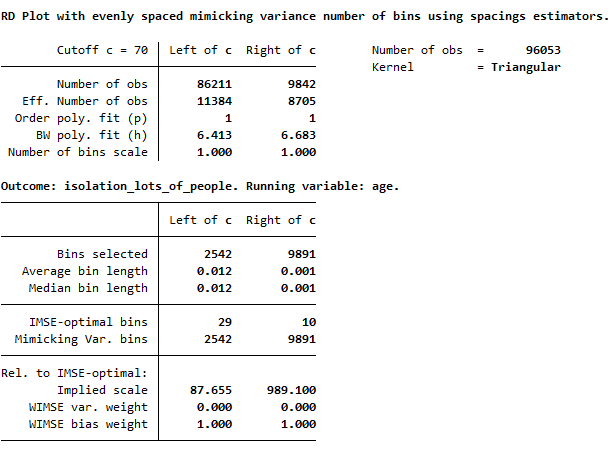

///Note: The other models are estimated in the same manner by changing the outcome variable. For subgroup analyses, we used if statements (e.g., if gender == 1 to subset the data to men). For quadratic models, we set p(2) instead p(1) in the rdrobust and rdplot functions. To adjust for covariates, we supplied covs(women risk1 risk2 any_lung risk4 risk7 risk9 in_sthlm) as an option for rdrobust.

## Density test

// 1. Plot a histogram of the running variable to assess jumps in the number of observations at the threshold (i.e., to assess manipulation/sorting around the threshold).

hist age, bin(40) xtitle("Age") xline(70) frequency

graph export "S_forcingvar_histogram.pdf", as(pdf) replace

//use rddensity to formally test for manipulation in the histogram at 70

rddensity age, c(70) nobinomial


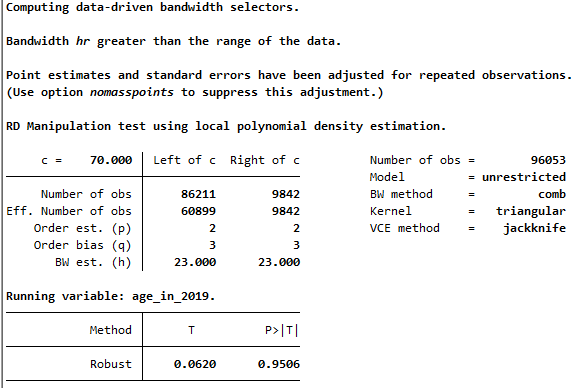


//Note: option c(70) tells the command that the cutoff is 70 years, and nobinomial suppresses output for an exact binomial test for regression discontinuity designs under a randomization assumption (not relevant to our data).

# References

1 Ludvigsson Jonas F, Andersson Eva, Ekbom Anders, Feychting Maria, Kim Jeong-Lim, Reuterwall Christina, et al. External review and validation of the Swedish national inpatient register. *BMC Public Health* 2011;**11**:450. Doi: 10.1186/1471-2458-11-450.

2 Brooke Hannah Louise, Talbäck Mats, Hörnblad Jesper, Johansson Lars Age, Ludvigsson Jonas Filip, Druid Henrik, et al. The Swedish cause of death register. *Eur J Epidemiol* 2017;**32**(9):765–73. Doi: 10.1007/s10654-017-0316-1.

3 Calonico Sebastian, Cattaneo Matias D., Titiunik Rocio. Robust Nonparametric Confidence Intervals for Regression-Discontinuity Designs. *Econometrica* 2014;**82**(6):2295–326. Doi: 10.3982/ECTA11757.

4 Cattaneo Matias D., Idrobo Nicolas, Titiunik Rocio. A Practical Introduction to Regression Discontinuity Designs: Foundations. *ArXiv:191109511 [Econ, Stat]* 2019. Doi: 10.1017/9781108684606.

5 Kolesár Michal, Rothe Christoph. Inference in Regression Discontinuity Designs with a Discrete Running Variable. *American Economic Review* 2018;**108**(8):2277–304. Doi: 10.1257/aer.20160945.

6 Lee David S., Card David. Regression discontinuity inference with specification error. *Journal of Econometrics* 2008;**142**(2):655–74. Doi: 10.1016/j.jeconom.2007.05.003.

7 Calonico Sebastian, Cattaneo Matias D., Farrell Max H., Titiunik Rocío. Rdrobust: Software for Regression-discontinuity Designs. *The Stata Journal* 2017;**17**(2):372–404. Doi: 10.1177/1536867X1701700208.

8 Hilton Boon Michele, Craig Peter, Thomson Hilary, Campbell Mhairi, Moore Laurence. Regression Discontinuity Designs in Health. *Epidemiology* 2021;**32**(1):87–93. Doi: 10.1097/EDE.0000000000001274.

9 McCrary Justin. Manipulation of the running variable in the regression discontinuity design: A density test. *Journal of Econometrics* 2008;**142**(2):698–714. Doi: 10.1016/j.jeconom.2007.05.005.

10 Cattaneo Matias D., Jansson Michael, Ma Xinwei. Manipulation Testing Based on Density Discontinuity. *The Stata Journal* 2018;**18**(1):234–61. Doi: 10.1177/1536867X1801800115.
